# Impaired barrier function by dietary fructo-oligosaccharides (FOS) in rats is accompanied by increased colonic mitochondrial gene expression

**DOI:** 10.1186/1471-2164-9-144

**Published:** 2008-03-27

**Authors:** Wendy Rodenburg, Jaap Keijer, Evelien Kramer, Carolien Vink, Roelof van der Meer, Ingeborg MJ Bovee-Oudenhoven

**Affiliations:** 1 TI Food and Nutrition, Wageningen, The Netherlands; 2 RIKILT – Institute of Food Safety, Wageningen, The Netherlands; 3 NIZO Food Research, Ede, The Netherlands; 4 Nutrigenomics Consortium, TI Food and Nutrition, Wageningen, The Netherlands

## Abstract

**Background:**

Dietary non-digestible carbohydrates stimulate the gut microflora and are therefore presumed to improve host resistance to intestinal infections. However, several strictly controlled rat infection studies showed that non-digestible fructo-oligosaccharides (FOS) increase, rather than decrease, translocation of *Salmonella *towards extra-intestinal sites. In addition, it was shown that FOS increases intestinal permeability already before infection. The mechanism responsible for this adverse effect of FOS is unclear. Possible explanations are altered mucosal integrity due to changes in tight junctions or changes in expression of defense molecules such as antimicrobials and mucins. To examine the mechanisms underlying weakening of the intestinal barrier by FOS, a controlled dietary intervention study was performed. Two groups of 12 rats were adapted to a diet with or without FOS. mRNA was collected from colonic mucosa and changes in gene expression were assessed for each individual rat using Agilent rat whole genome microarrays.

**Results:**

Among the 997 FOS induced genes we observed less mucosal integrity related genes than expected with the clear permeability changes. FOS did not induce changes in tight junction genes and only 8 genes related to mucosal defense were induced by FOS. These small effects are unlikely the cause for the clear increase in intestinal permeability that is observed. FOS significantly increased expression of 177 mitochondria-related genes. More specifically, induced expression of genes involved in all five OXPHOS complexes and the TCA cycle was observed. These results indicate that dietary FOS influences intestinal mucosal energy metabolism. Furthermore, increased expression of 113 genes related to protein turnover, including proteasome genes, ribosomal genes and protein maturation related genes, was seen. FOS upregulated expression of the peptide hormone proglucagon gene, in agreement with previous studies, as well as three other peptide hormone genes; peptide YY, pancreatic polypeptide and cholecystokinin.

**Conclusion:**

We conclude that altered energy metabolism may underly colonic barrier function disruption due to FOS feeding in rats.

## Background

Non-digestible carbohydrates like fructo-oligosaccharides (FOS) stimulate the gut microflora and are therefore presumed to improve host resistance to intestinal infections. For this reason non-digestible carbohydrates are added to a growing list of products, including baby-formula, bread, dairy products. Many studies, including our own, showed that non-digestible carbohydrates indeed affect intestinal microflora composition [1-3]. However, there is little evidence that these non-digestible carbohydrates strengthen intestinal resistance to infection and gut barrier function.

For this reason, several strictly controlled rat infection studies were previously performed at our lab. These studies consistently showed that the non-digestible carbohydrates inulin, lactulose and FOS increase translocation of *Salmonella *to extra-intestinal organs [3-5]. A dose-dependent increase in *Salmonella *translocation was observed in FOS-fed rats [3]. Stimulation of *Salmonella *translocation by dietary FOS was reflected in transcriptional changes in colon. Genes involved in antimicrobial defense, immune response and inflammation were induced by *Salmonella *infection of rats on a control diet and further upregulated in *Salmonella *infected rats on a FOS diet [6]. Moreover, intestinal barrier parameters were already affected by FOS before infection. In particular intestinal permeability was increased by FOS before *Salmonella *challenge. Also, luminal cytotoxicity and faecal mucin excretion were increased in FOS-fed rats and may indicate mucosal irritation [5].

The intestinal barrier is mainly formed by the mucosal epithelial lining. Disturbed barrier function can be monitored by measurement of epithelial permeability using inert permeability markers such as different kind of sugars or CrEDTA [7,8]. It has been shown that increased transport of large molecules or antigens due to increased permeability may initiate inflammation [9]. Several mechanisms have been implicated in the mucosal barrier. Tight junctions tightly connect the epithelial cells and regulate paracellular transport of fluids, electrolytes and small compounds [10]. Modification or cellular translocation of the tight junction molecules ZO1 and several claudins have indeed been observed in inflammatory bowel disease (IBD) and chronic non-steroidal anti-inflammatory drugs (NSAIDs) use, both characterized by increased intestinal permeability [11,12].

In addition to changes in epithelial tight junctions, balance between apoptosis and proliferation, or regeneration, is also a major determinant of an intact mucosal epithelial lining [13]. Increased apoptosis can induce epithelial leakage as shown in colonic epithelial cell lines and in intestinal biopsy specimens of IBD patients [14].

Apart from tight junctions and apoptosis, secretory products of intestinal epithelial cells are known to play a role in mucosal barrier. The different intestinal epithelial cells; enterocytes, goblet cells, Paneth cells and enteroendocrine cells, are equipped with defense mechanisms. For instance Paneth cells in the crypt base produce antimicrobials, such as defensins, lysozyme and Pla2g2a [15], to regulate and restrict the bacterial load in the gut lumen. Goblet cells produce mucins to form a mucus layer, this layer functions as a physical barrier that protects the epithelial cells from harmful compounds [16]. Furthermore, the immune system plays an important role in mucosal integrity [17]. Whether other processes are involved and the relative importance of these mechanisms for intestinal barrier integrity is not known. Also it is not known if any of the above mentioned mechanisms or others are responsible for the effect of FOS on gut permeability.

Therefore, we analyzed colonic gene expression changes in individual rats fed a 6% FOS diet for 2 weeks. Analyzing FOS induced gene expression using whole genome microarrays allowed us to not only focus on the above mentioned mechanisms, but to obtain an unbiased view on processes affected by dietary FOS. This facilitates identification of genes and processes currently unknown to be related to barrier function. The colon had our main interest as FOS and other non-digestible carbohydrates are exclusively fermented in the distal gut in humans [18] and rats [3]. We aimed to identify the *in vivo *biological mechanisms involved in FOS-induced weakening of the barrier in rats.

## Results

### Food intake, body weight gain, and intestinal permeability

**Figure 1:**
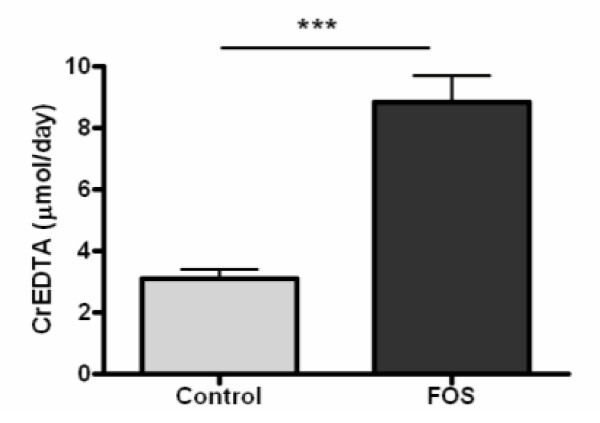
Average urinary CrEDTA excretion in the control and FOS group. Daily dietary CrEDTA intake was 54 μmol. Urines were collected at days 14 and 15. Results are expressed as mean ± SEM (n = 6 per diet group). The FOS group significantly differed from the control group (***p < 0.001).

Rats on the control diet and FOS diet showed no significant difference in body weight gain. Both dietary groups consumed the provided 14 grams of diet per day as intended and thus had a similar dietary CrEDTA intake. Intestinal permeability was examined by measurement of CrEDTA excretion in urine and showed that FOS fed rats had increased urinary excretion of this inert permeability marker as compared to the control group (Figure 1).

### Gene expression profile

28180 probes on the array had an expression value of 1.5 times above background. Of these 123 were induced and 56 were reduced more than 1.5 fold in FOS fed rats compared with rats fed the control diet. Only 19 probes were induced more than 2-fold. While gene expression changes induced by FOS supplementation were small, there were many significantly changed probes in the dataset: 803 had a p-value < 0.001, and 231 probes had a FDR derived q-value < 0.01. This indicated that the gene expression response of colon mucosa to FOS was small in magnitude, but highly significant. As each probe signal is tested independently in t-test and multiple testing increases the risk for finding false positives, we also applied Random Forest [19,20]. RF ranks genes based on an importance value taking main treatment effect as well as gene-gene interaction into account [20]. Using RF, we identified 935 probes with an importance value above threshold. 629 were selected by both the t-test and RF. To prevent loss of information, we used all genes selected by t-test and RF. This resulted in a list of 1109 probes. 112 corresponding genes were listed more than once. Therefore, duplicates with the highest p-values were removed, resulting in 997 unique genes regulated by FOS

Gene selection by t-test or RF alone substantially overlapped and did result in a highly comparable outcome in pathway analysis, showing that the main effects extracted by both methods were similar. However, the individual gene selection differed slightly between both methods. We choose to include all genes selected by either method.

### Effect of FOS on the expression of barrier associated genes

As FOS affects the mucosal barrier, we specifically analyzed genes that are known or assumed to play a role in barrier function. These include tight junction genes like *Zo-1*, occludin and claudin, cell turnover/apoptosis genes such as caspases, *Bak, Bcl-2*, and mucosal defense genes such as defensins, lipocalin, toll like receptors and IgA (Additional file 1). Tight junction related genes were not affected by FOS. Several apoptosis (for example *Bax, DNase1, Pdcd 6 & 8*) and mucosal defense genes (for example phospholipase A2 and trefoil factor 1 & 3) were increased by FOS (Additional file 1). However, no FOS effect was found on other mucosal defense genes like IgA, Mucin 2 & 3, defensins, lipocalin, calprotectin, and most toll-like receptors. In addition, some markers of apoptosis were slightly affected (Bak, Caspase 7), while most (including Apaf, Caspase 9, caspase 3, caspase 2, Bcl2 and Bad) were not affected by FOS.

### Genes most prominently affected by FOS

We examined the top 10 of genes most affected by the FOS diet (Table 1). We choose the genes most prioritized by RF. These genes were characterized by extremely low p-values and relatively high fold changes. The genes were related to nutrient homeostasis (proglucagon), energy metabolism (NADH dehydrogenases (*Ndufb6, Ndufa4 *and *Ndufb5*) and ATP synthase (*Atp5f1*)), protein turnover (Proteasome subunit alpha type 3-like (*Psma3l*)), oxidative stress response (Metallothionein-2 (*Mt-2*)) and retinol metabolism (cellular retinol-binding protein (*Rbp7*)). This top-10 list indicates that FOS especially affects cellular energy metabolism in rat colonic mucosa, this was supported by the pathway analysis results, as described below.

**Table 1:** Top 10 of highest ranked genes by t-test and Random Forest.

Gene name	Sequence ID	Gene Symbol	Fold change*	p value
Glucagon gene, exon 6	K02813	*Glc*	2.6	5E-10
Cellular retinol-binding protein 7	P02696	*Crbp*	4.0	2E-09
NADH dehydrogenase (ubiquinone) 1 beta subcomplex, 5	XM_215544	*Ndufb5*	1.3	3E-08
Unknown (LOC295337)	XM_215660	*-*	1.6	4E-08
NADH dehydrogenase (ubiquinone) 1 beta subcomplex, 6	XM_216378	*Ndufb6*	1.5	7E-08
ATP synthase, H+ transporting, mitochondrial F0 complex, subunit b, isoform 1	NM_134365	*Atp5f1*	1.4	3E-07
Protein C11orf10, LOC309206	XM_219574	*-*	1.4	3E-07
NADH dehydrogenase (ubiquinone) 1 alpha subcomplex, 4	NM_010886	*Ndufa4*	1.4	3E-06
Metallothionein-2	BF556648	*Mt2*	1.9	4E-06
Proteasome subunit alpha type 3-like	BN000326	*Psma3l*	1.4	7E-06

*Fold change FOS/control

### Pathway analysis

Pathway analysis identified processes most affected by dietary FOS in colonic mucosa. 366 Genes of the 997 selected genes could be classified based on GO term or based on GeneGo annotation in the Metacore database [21]. The most significant processes were an entire range of mitochondria related processes such as mitochondrial electron transport, oxidative phosphorylation, translation in mitochondria and proteins targeted to mitochondria (Additional file 2). Another highly classified process was proteolysis (Additional file 2). To prevent bias in biological interpretation due to gene selection (by t-test and RF), we also applied Gene Set Enrichment Analysis (GSEA) which includes all genes in the dataset (28180 genes). We focused on curated gene-sets originating from GenMapp, Biocarta, SigmaAldrich and Broad institute. Comparable biological processes were found by GSEA as observed in Metacore: again oxidative phosphorylation was most significant, followed by proteasomal degradation (Additional file 3). Thus the threshold based Metacore analysis and the threshold free enrichment analysis, GSEA, gave similar results for the most significantly changed processes by FOS. The results obtained by pathway programs consist of many overlapping pathways. We combined pathways with overlapping genes such as mitochondrial electron transport (Metacore), electron transport (Metacore) and electron transport chain (GSEA) and categorized the processes.

**Figure 2:**
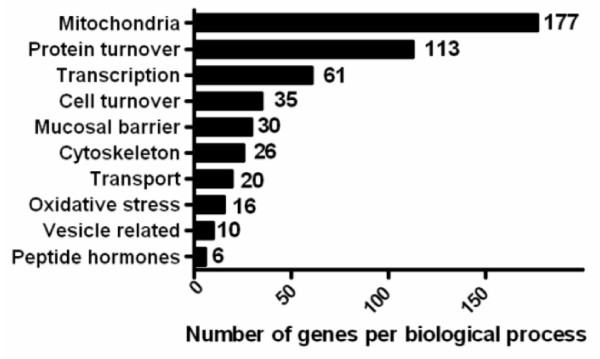
Classification of the genes affected by FOS into biological processes. Analyzed by Metacore, GSEA and data mining.

Analysis based on pathway programs is restricted to the well annotated genes [22]. As only 36% of the Agilent whole genome array is recognized by Metacore and only 35% by GSEA, we manually extended the significantly altered pathways with the non-recognized genes using literature and databases mining (using Biocarta, Source, Genecards). This strengthened the pathway outcome, as we were able to identify many additional genes affected by FOS that could be added to the processes already identified by the pathway programs. This was the case for transcription identified by Metacore as nucleosome assembly, cell turnover identified by GSEA as programmed cell death, cytoskeleton and vesicle related processes (muscle filament sliding and cytoskeleton-dependent intracellular transport in Metacore) and oxidative stress (free radical induced apoptosis in GSEA) (Figure 2). In addition, we identified FOS affected genes that were not grouped into a pathway by both programs but obviously belong to the same biological process, this was the case for mucosal barrier, transport, and peptide hormones (Figure 2).

Detailed analysis of the mitochondrial processes showed an increased expression of genes associated with al five complexes of the OXPHOS complex, TCA-cycle and mitochondrial ribosomes and mitochondrial protein transport (Table 2). In addition to protein degradation, which was found in the pathway programs, protein translation and maturation were also affected by FOS. From these 113 genes more than 90% showed increased expression upon FOS indicating increased protein turnover (Table 2). Detailed gene expression data for all processes mentioned in Table 2 is presented in Additional file 4.

**Table 2:** Detailed classification of biological processes affected by FOS.

Biological process		Number of genes affected by FOS*
*Mitochondria*	Complex I	27
	Complex II	5
	Complex III	2
	Complex IV	13
	Complex V	21
	Metabolism and TCA cycle	53
	Mitochondrial ribosomes	33
	Protein transport	11
	Miscellaneous	12
*Protein turnover*	Protein degradation	31
	Translation	44
	Protein maturation	32
	Miscellaneous	6
*Transcription*	Chromatin related	22
	mRNA metabolism	7
	Transcription	26
	Miscellaneous	6
*Cell turnover*	Apoptosis	19
	Growth/Differentiation	16
*Mucosal barrier*		30
*Cytoskeleton*		26
*Transport*		20
*Oxidative stress*		16
*Vesicle related*		10
*Peptide hormones*		6
*Other†*		503
		997

* Genes with p-value < 0.001 or selected by RF threshold

† Genes with less than 5 other genes belonging to the same process, unknown genes and, genes not part of a known process.

### Confirmation of array results by Q-PCR

Confirmation of FOS induced processes was performed by Q-PCR. Genes from several FOS affected processes were analyzed by individual Q-PCR. We selected nine genes from mitochondria related processes: NADH dehydrogenase (ubiquinone) 1 beta subcomplex 9 (*Nduf9b*), succinate dehydrogenase complex subunit B (*Sdhb*), ubiquinol-cytochrome c reductase binding protein (*UbiqcytC*), cytochrome c oxidase subunit VIIb (*Cox7b*), ATP synthase H^+ ^transporting mitochondrial F0 complex subunit G (*ATP5g*), aldo-keto reductase family 1 member B8 (*Akr1b8*), malic enzyme 1 (*Me1*), mitochondrial ribosomal protein S16 (*RiboS16*), translocase of inner mitochondrial membrane 8 homolog b (*Timm8b*). In addition, one gene from protein degradation proteasome subunit alpha type 3-like (*Psma3l*), two genes related to mucosal barrier phospholipase A2, group IIA (platelets synovial fluid) (*Pla2g2a*) and trefoil factor 3 (*Tff3*) and one gene from the peptide hormones, proglucagon (*Gcg*) were analyzed. We additionally selected retinol binding protein 7 (*Rbp7*) as this gene was highest induced by FOS (4-fold). Q-PCR fully confirmed the microarray data (table 3). The p-value in the Q-PCR analysis reached significance (p < 0.05) for 11 out of 13 genes. Two genes had a p-value > 0.05 (*ATP5g *and *Timm8b*).

**Table 3:** Q-PCR confirmation of microarray data.

			Micro-array	Q-PCR		
				
Gene name	Gene symbol	Sequence ID	Ratio*	Ratio*	SEM ±	p-value†
**Mitochondria**						
*Complex I*						
NADH dehydrogenase (ubiquinone) 1 beta subcomplex, 9	*Nduf9b*	XM_216929	1.47	1.29	0.05	0.001
*Complex II*						
Succinate dehydrogenase complex, subunit B, iron sulfur (Ip)	*Sdhb*	XM_216558	1.43	1.32	0.05	<0.001
*Complex III*						
Ubiquinol-cytochrome c reductase binding protein	*Uqcrb*	XM_001074024	1.53	1.28	0.05	0.005
*Complex IV*						
Cytochrome c oxidase subunit VIIb	*Cox7b*	NM_182819	1.61	1.36	0.06	0.001
*Complex V*						
ATP synthase, H+ transporting, mitochondrial F0 complex, subunit G	*ATP5g*	XM_001075306	1.52	1.15	0.05	0.11
*Metabolism and TCA cycle*						
Aldo-keto reductase family 1, member B8	*Akr1b8*	NM_173136	2.13	2.15	0.22	0.006
Malic enzyme 1	*Me1*		1.65	1.76	0.11	<0.001
*Mitochondrial ribosomes*						
Mitochondrial ribosomal protein S16	*Mrps16*	XM_001064095	1.43	1.20	0.05	0.03
*Mitochondrial protein transport*						
Translocase of inner mitochondrial membrane 8 homolog b	*Timm8b*	NM_022541	1.45	1.19	0.06	0.09
**Protein turnover**						
Proteasome subunit alpha type 3-like	*Psma31*	BN000326	1.39	1.24	0.04	0.002
**Mucosal barrier**						
Phospholipase A2, group IIA (platelets, synovial fluid)	*Pla2g2a*	NM_031598	3.73	4.70	0.87	0.03
Trefoil factor 3	*Tff3*	NM_013042	1.7	1.21	0.05	0.04
**Top 10 gene**						
Retinol binding protein 7	*Rbp7*	XM_575960	2.06	3.72	0.36	<0.001
**Peptide hormone**						
Proglucagon	*Gcg*	NM_012707	2.56	2.91	0.23	<0.001

* Ratio FOS diet/Control diet.

± SEM of Q-PCR data is given (n = 12 per group)

† p-value of Q-PCR data is given, the p-value of microarray data were all <0.001.

### Confirmation of gene expression results on protein level

**Figure 3:**
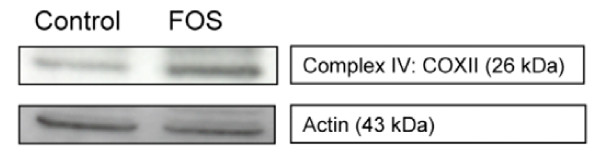
Mucosal scrapings (pool of n = 12 per group) were examined for complex IV subunit COXII protein levels. The experiment was performed three times with independent, pools, showing a 1.5; 1.7 and 2.7 fold difference in COXII protein expression relative to Actin, respectively. The 1.5 fold increase is shown.

To substantiate the FOS induced transcriptional modulation of mitochondrial genes at the protein level, we analyzed pooled mucosal scrapings of all rats (n = 12 per group). The small increase in mRNA levels of complex IV subunits (~1.4 fold), was confirmed by a similar increase (1.5, 1.7 and 2.7 fold in independent pools, relative to actin) in protein levels of complex IV subunit COX II in the FOS group compared with the control group in pooled (n = 12) mucosal scrapings (Figure 3).

**Figure 4:**
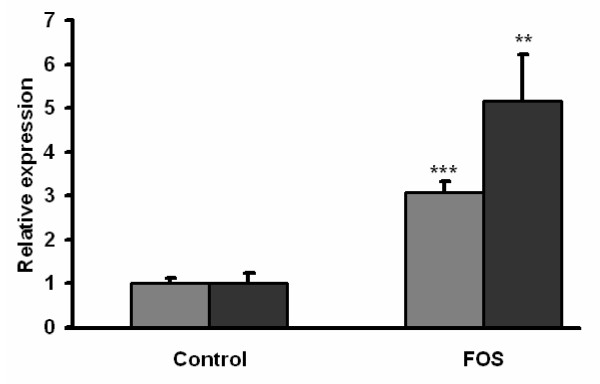
Relative expression of Proglucagon mRNA (gray bar) and GLP-1 protein (black bar) in colonic mucosa of a random selection of control fed and FOS fed rats. mRNA and protein levels were normalized to Actin levels. Expression is shown as means ± SEM (n = 7). **p < 0.01, ***P < 0.001

The relatively high and significant induction of proglucagon gene by FOS was also examined at the protein level. Mucosal scrapings of randomly sampled control and FOS-fed rats (n = 7 per group) were analyzed for GLP-1 protein levels by western blot and normalized to actin. FOS significantly increased GLP-1 protein levels in colonic mucosal tissue (Figure 4), substantiating the gene expression findings.

## Discussion

The CrEDTA results showed that dietary FOS increased intestinal permeability in rats in accordance with our previous study [5]. The FOS induced increase in intestinal permeability reduces intestinal barrier function as reported earlier [5]. We focused on the colon since fermentation of FOS occurs in cecum and colon and is very limited in the ileum [18,23]. Furthermore, by additional use of lactulose/mannitol as permeability labels [24] we found that FOS increased intestinal permeability in the large intestine and not in the small intestine (unpublished results). Individual colonic gene expression of 12 rats after ≈2 weeks FOS versus control feeding were explored on whole genome level and showed that the increased permeability could not be explained by changes in genes belonging to the tight junction system in the colon. No significant changes were observed in claudin 2 and 4, cadherins or tight junction protein 1. With our focus on gene expression, possible changes in protein levels and cellular localization or modification cannot be excluded. 19 Genes related to apoptosis were affected by FOS with only modest fold changes. Although some pro-apoptotic genes were mildly affected (eg Bax, Caspase 7), no changes were seen in many key pro-apoptotic genes such as Bad, Caspase 3 and Apaf1. Therefore, we feel that apoptosis is not the main cause of the increased intestinal permeability observed. Known mucosal defense genes such as defensins, mucins and calprotectin were also unaffected by FOS. These few and small transcriptional changes in potential barrier related genes cannot explain the profound and consistent effects of FOS on intestinal permeability in rats. We can not fully exclude that dilution of specific cell types in the heterogeneous cell population of mucosal scrapings has lead to undetectable levels of some expected genes, e.g. mucin genes. This could be further assessed by laser microcapture dissection, which allows isolation of specific cell types from the intestine [25], and subsequent gene expression analysis by Q-PCR. On the other hand, we identified multiple genes associated with energy metabolism (177 mitochondria related genes) that were significantly modified by FOS. Protein turnover was also clearly affected by FOS (113 genes). Coincidence of increased permeability and upregulation of these genes suggests that these processes play a major role in preservation of intestinal mucosal integrity.

The most striking observation was the induction of a broad range of mitochondrial genes. Increased expression of mitochondrial genes is commonly observed during disturbed ATP homeostasis caused by increased energy demand or decreased mitochondrial energy supply [11-14]. Most of the genes reported to be changed by disturbed ATP homeostasis were also induced by FOS (*Ant*, 24 NADH dehydrogenases, 7 *Cox *subunits and 3 ATPases). This could be confirmed at protein level for complex IV subunit II. Together this strongly suggests that FOS caused ATP depletion in colonic epithelial cells.

The increase in mitochondrial genes could indicate a compensation for possible SCFA – induced ATP depletion. The validity of this assumption could be studied by measuring ATP levels in enterocytes of FOS- and control-fed rats. The clear changes of FOS on mitochondrial processes were not expected beforehand, therefore no precautions were taken at the time of sampling and storage of the mucosal scrapings that would allow post-hoc analysis of ATP levels. Alternatively we attempted to examine levels of phosphorylated AMP-activated protein kinase (AMPK) as this reflects the ADP/ATP ratio in cells [26]. Low levels of total AMPK protein could be detected in colonic scrapings without difference between control and FOS (data not shown). However, AMPK phosphorylation could not be detected in mucosal scrapings, most certainly because of instability of the phosphorylated protein requiring specific sample collection procedures. In addition it would be interesting to study the effects of FOS on mitochondria using electron microscopy studies [27].

So, the question remains whether the strongly and broadly increased expression of mitochondrial genes by FOS, is associated with the observed increase in intestinal permeability in FOS-fed rats. Many studies using epithelial cell lines have shown that paracellular hyperpermeability is caused by ATP depletion [28-30]. Another strong indication that mitochondria are important in maintaining intestinal permeability is derived from studies on chronic NSAIDs [31]. Direct exposure of rat intestine to the NSAID indomethacin or the mitochondrial uncoupler DNP increases epithelial permeability [32-34], which increased bacterial translocation in rats and intestinal cell lines, and increased immune cell infiltration and ulceration in rats [32,35]. Enhanced bacterial translocation reflects impaired barrier function.

**Figure 5:**
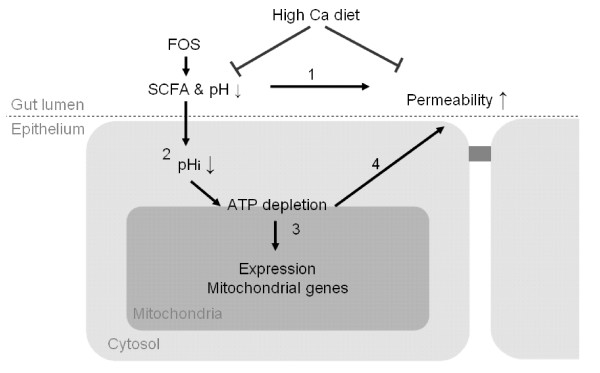
**Proposed mechanism of dietary FOS induced intestinal permeability**. 1 High levels of FOS fermentation products increase intestinal permeability *in vivo *[5, 40, 41]. 2 Excess SCFAs cause intracellular acidification of epithelial cells. When protonated-SCFA diffuse from the gut lumen into epithelial cells [47, 84]. The SCFA cause intracellular acidification and induce proton pump activity (NHE and NBC transporters) which may lead to ATP depletion [43, 45]. 3 Reduced ATP levels, by increased energy demand, chronic mitochondrial uncoupling or any other cause of disturbed energy metabolism, are compensated by increased mitochondrial gene expression and mitochondrial biogenesis [27, 85]. 4 Disturbed energy metabolism leads to increased permeability. In agreement: ATP-depletion in epithelial cell lines causes paracellular hyperpermeability [28-30] and uncoupling of intestinal mitochondria leads to increased bacterial translocation, immune cell infiltration and ulceration in rats [31, 32]. Calcium supplementation of a FOS diet counteracts FOS induced intestinal permeability. Calcium prevents acidification of intestinal contents during fermentation and thus formation of protonated-SCFA.

In Figure 5, we schematically describe a possible mechanism of the FOS induced increase in intestinal mitochondrial gene expression, the proposed ATP-depletion and the increased mucosal permeability observed.

Previous studies in rats [3,36,37] and humans [38] showed rapid fermentation of FOS in cecum and colon by the endogenous microflora resulting in lactic acid accumulation, SCFA production, and decreased pH of luminal contents. Although luminal production of modest quantities of SCFAs is essential for normal colonic mucosal function [39], overproduction or accumulation of SCFA along with low pH in the intestinal lumen has been shown to cause intestinal injury leading to increased intestinal permeability *in vivo *[40-42] and *in vitro *[43,44]. Studies in liver show that SCFA induced depletion of cellular ATP coincided with a reduced intracellular pH (pH_i_) [45]. A likely explanation for reduced pH_i _by SCFA is that in an acidic luminal environment, a relatively larger portion of SCFA becomes protonated, facilitating passive diffusion of SCFA across the apical hydrophobic enterocyte membrane causing intracellular acidification [46,47]. The above mentioned studies were done *in vitro *or in perfused liver, but the concentrations applied can impair the pH_i _homeostasis in colonocytes in FOS fed rats [36,48]. Taken together, FOS reduce luminal pH and increase levels of SCFAs, likely leading to acidification of the cellular cytoplasm (decrease in pH_i_). A decrease in pH_i _is compensated by H^+ ^excretion in exchange for Na^+ ^by the pH_i _regulating Na^+^/HCO3^- ^cotransporter (NBC) and the Na^+^/H^+ ^exchanger (NHE) in the basolateral membrane [49,50]. This indirectly activates the Na+,K+-ATPase (ATP1) which is known to require ~25% of the cellular ATP turnover under basal conditions [51]. Long-term exposure of enterocytes to high SCFA concentrations under low pH conditions might therefore disturb or exhaust plasma membrane pumps, leading to rapid ATP-depletion [43]. FOS diet significantly increased expression of *Atp1b1 *(1.2-fold, p < 0.001). No changes were found in the gene expression of the transporters *Nbc *and *Nhe*, but increased activity of these transporters can occur without concomitant changes in mRNA gene expression.

A strong indication supporting SCFA and low luminal pH as inducers of the observed effects, are results of previous studies of our lab showing that the adverse affects of FOS on mucosal barrier, i.e. increased permeability and decreased resistance to pathogens, are absent when calcium is supplemented to the diets [4]. Calcium is known to increase the buffering capacity of luminal contents by its precipitation with dietary phosphate. By preventing acidification during fermentation, SCFA will remain in the anionic form, for which the cellular plasma membrane is not permeable. Other possible mechanisms may play a role, including SCFA induced accumulation of Acyl CoA in mitochondria, opening of the pore complex or modulation of histone deacetylase activity by butyrate followed by an altered PGC1 expression, but none of these mechanisms were supported by consistent changes in gene expression.

The second major process affected by FOS was protein turnover. FOS induced 27 ribosomal proteins and 30 proteasomal genes, indicating increased protein turnover. Intracellular proteins are targeted to the proteasomal degradation system by ubiquitination [52]. Proteasomal degradation is tightly controlled and removes denatured, misfolded and damaged proteins. The clear increase in proteasomal gene expression might result from increased presence of misfolded proteins. One common cause of misfolding of cellular proteins is mild oxidative stress [53]. FOS induced several genes related to oxidative stress, such as metallothionein-2 (*Mt2*) metallothionein-1a (*Mt1a*), six glutathione S-transferases (*Gst*'s), heme oxygenase 1 (*Hmox1*), and superoxide dismutase 1 and 3 (*Sod1 *and *Sod3*). An increase in oxidative stress proteins is an indirect marker for production of reactive oxygen species (ROS) [54]. It is well recognized that induced mitochondrial activity can increase production of ROS. The increased expression of oxidative stress genes and mitochondrial genes observed in the present study suggests increased mitochondrial activity, possibly associated with increased ROS production and increased protein oxidation. Oxidized proteins are often misfolded, and directed to proteasomes for degradation. In support, the translocase *Sec61 *responsible for intracellular transport of misfolded proteins from the ER to the proteasome was significantly increased 1.4 fold (p < 0.001) by FOS.

Increased proteasomal gene expression might also reflect the formation of immunoproteasomes (I-proteasomes) [55]. The I-proteasome plays a role in antigen processing and is composed of two subunits PSME1 and PSME2 [55] which were both significantly increased by FOS supplementation. Expression of these subunits is known to be induced by microbial infection and cytokines like IFNγ [56]. Alterations in the I-proteasome in the injured intestinal epithelium are observed in colon biopsies of IBD patients and IBD-mouse models [55,57]. The increased intestinal permeability due to the FOS diet can cause increased exposure of the mucosa to bacteria and therefore induce I-proteasome gene expression.

The top 10 most significantly changed genes by FOS showed to be good representatives of the major biological processes selected from pathway analysis. In addition to genes related to mitochondria and protein turnover, two genes coding for cellular retinol-binding proteins (*Rbp7 and Rbp2*) were highly induced by FOS. RBPs are required for uptake, intracellular transport and metabolism of vitamin A. Vitamin A is a fat-soluble vitamin necessary for growth and differentiation of epithelial tissues. RBP7 and RBP2 belong to the fatty-acid binding protein (FABP) family. FOS also significantly increased *Fabp1 *2.9 fold. At present, we cannot explain the FOS induced expression of these genes and its relation to the functional effects observed in this and our earlier FOS studies.

Proglucagon is one of the most highly induced gene by FOS (2.6-fold, p < 0.001). The proglucagon gene is a precursor encoding several glucagon-like peptides. In intestinal enteroendocrine cells the gene codes for oxyntomodulin, GLP1 and GLP2 [58]. This gene was previously found to be induced by non-digestible oligosaccharides [59]. Besides FOS induced expression of proglucagon, FOS induced the expression of several other gut-derived peptide hormones, namely cholecystokinin (*Cck*), peptide YY (*Pyy*) and pancreatic polypeptide (*Ppy*). Increase of PYY has previously been reported in rat colon by SCFA [60]. Proglucagon, PYY, PPY and CCK are all expressed by enteroendocrine L cells in colon and play a role in gut-nutrient sensing [61]. In the hypothalamus nutrient sensing is also regulated by these hormones and directly related to ATP status [62]. It is speculated that the same mechanism is applicable to enteroendocrine L cells in de gut [60]. Since in our study, increased gene expression of these 4 peptide hormone genes coincides with alterations in mitochondrial processes, it is tempting to speculate that these hormones also influence or respond to energy metabolism in intestinal epithelial cells. Besides a role in energy homeostasis, GLP-2, PYY and CCK have growth-promoting properties on the intestinal epithelium *in vivo *[63]. GLP1 stimulated cell proliferation has been reported for liver and pancreas [64]. GLP2 is involved in regulation of mucosal epithelial integrity) [65]. It stimulates intestinal crypt cell proliferation [66] and reduces apoptosis, therefore enhances mucosal regeneration. It has beneficial effects on many causes of intestinal injury, such as stress, vascular ischemia, NSAID administration and chemically induced injury in rodents, and decreases subsequent intestinal permeability [67,68]. The upregulation of these genes might thus be a response to the impaired intestinal barrier in FOS-fed rats.

FOS consistently increased intestinal permeability, but the present study showed hardly any effect on expression of well known intestinal integrity genes in the colon. Most surprisingly no changes were observed in genes related to tight junctions that were expected since tight junctions are key regulators of paracellular transport. However, changes in epithelial permeability are a result of internalization of the tight-junction proteins occludin, claudin and junctional adhesion molecule-A [69]. These cellular translocations can occur without concomitant changes in mRNA gene expression. Detection of such effects would require a different approach from transcriptomics. Immunohistochemistry could show whether translocation of TJ proteins occurred in the FOS fed rats compared with control fed rats and is under current investigation.

## Conclusion

Altogether we show that altered barrier integrity induced by dietary FOS in rats coincides with a clear increase in colonic mitochondrial gene expression, suggesting that mitochondrial energy metabolism is important for maintaining the intestinal barrier. The role of mitochondria in maintenance of the intestinal barrier is accepted in NSAID and DNP uncoupling studies. We speculate that FOS induced excess production of SCFA and acidification of luminal contents might result in SCFA induced ATP depletion of colonic epithelial cells. Insight into the role of mitochondrial function and ATP depletion is of relevance, not only for the application of FOS and other prebiotics in food products on the current market, but especially for mechanistic understanding of intestinal disorders where gut permeability changes are observed.

## Methods

### Animals and diet

The animal welfare committee of Wageningen University (Wageningen, the Netherlands) approved the experimental protocol. Specific pathogen-free male outbred Wister rats (8 weeks old, mean body weight of 253 g; n = 36 in total), were housed individually in metabolic cages. All animals were kept in a temperature (22–24°C) and humidity (50–60%) controlled room with a 12 h light/dark cycle (lights on from 6 AM to 6 PM). Rats (two dietary groups, n = 18 each) were fed restricted quantities (14 g/day) of a purified diet during the entire experimental period. Restricted food intake was necessary to prevent differences in food consumption and hence differences in vitamin and mineral intake as observed earlier in FOS interventions [3]. The diet contained (per kg) 200 g acid casein, 502 g glucose, 160 g palm oil, 40 g corn oil, 20 g cellulose, 35 g mineral mix (without calcium) and 10 g vitamin mix according to AIN93 recommendations [70]. Diets contained 20 g/kg cellulose at least and were supplemented with either 60 g/kg FOS (purity 93%; Raftilose P95, Orafti, Tienen, Belgium) or additional 60 g/kg cellulose as described earlier [3]. Diets were low in calcium (20 mmol CaHPO_4_.2H_2_O/kg) and high in fat content (200 g fat/kg) to mimic the composition of a Western human diet. Demineralized drinking water was supplied *ad libitum*.

To follow intestinal permeability, 6 of the 18 rats of each dietary group received their diet supplemented with the intestinal permeability marker chromium ethylenediamine-tetraacetic acid (CrEDTA). The CrEDTA solution added to the diet was prepared as described elsewhere [5]. After feeding the diets for 16 days, rats were killed by carbon dioxide inhalation. Rats fed diets containing the permeability marker CrEDTA (n = 6 per diet group) were not included in the gene expression study, to exclude possible interaction of CrEDTA on colonic gene expression. From the remaining 12 rats per dietary group, the colon was taken out, longitudinally opened and colonic contents were removed by a quick rinse in 154 mM KCl. Colonic mucosa was scraped off using a spatula. Scrapings were immediately frozen in liquid nitrogen and stored at -80°C. The scrapings were homogenized in liquid N_2 _using a mortar and pestle cooled with liquid N_2 _(Fisher Emergo, Landsmeer, The Netherlands). One third of the pulverized samples was used for protein determination and the remaining part for RNA isolation.

### Analysis of urine samples

Total 24 h urine samples were collected on days 14 and 15 from rats fed the control and FOS diet (n = 6 each) supplemented with CrEDTA. Urines were preserved by adding oxytetracycline (1 mg) to the urine collection vessels of the metabolic cages, and analyzed for the intestinal permeability marker CrEDTA as described elsewhere [5]. CrEDTA data were analyzed using the Student's t-test (two-sided) using Prism 4 (GraphPad software Inc., San Diego, CA).

### RNA isolation

Total RNA was isolated from colon scraping homogenates using TRIzol reagent (Invitrogen, San Diego, CA) according to the manufacturer's instructions. Total RNA was purified using Rneasy columns (Qiagen, Chatsworth, CA). Absence of RNA degradation was checked on a 1% TBE/agarose gel after 1 hour incubation at 37°C. RNA purity and concentration were measured with the Nanodrop (Isogen Life Science, Maarssen, The Netherlands). OD A_260_/A_280 _ratios were all between 2.08 and 2.10 indicating RNA of high purity.

### Analysis of mRNA expression by Oligo Arrays

For microarray hybridization, RNA of each individual animal was labeled with Cy-5. A standard reference sample, consisting of a pool of all colonic RNA was labeled with Cy-3. For each oligo array, 35 μg of total RNA was used to make Cy-5 or Cy-3 labeled cDNA. Total RNA was mixed with 4 μg T21 primer, heated at 65°C for 3 min (RNA denaturation) followed by 25°C for 10 min (primer annealing). cDNA was synthesized by adding 5× first strand buffer (Invitrogen), 10 mM DTT, 0.5 mM dATP, 0.5 mM dGTP, 0.5 mM dTTP, 0.04 mM dCTP, 0.04 mM Cy5-dCTP or Cy3-dCTP, 1.2 U RnaseOUT and 6 U SuperScript II Reverse Transcriptase to a total volume of 62.5 μL. The reaction was incubated at 42°C for 2 h. Purification, precipitation and denaturation of the labeled cDNA were performed as previously described [71].

Each labeled cDNA sample was individually hybridized on the 44 K rat whole genome Agilent array (G4131A, Agilent Technologies, Inc. Santa Clara, CA) consisting of 44290 60-mer rat oligonucleotide probes, including ~3000 control spots. The Cy5 labeled cDNAs of the individual rats were mixed 1:1 with the Cy3 labeled reference cDNA, mixed with 2× hybridization buffer (Agilent Technologies) and 10× control targets (Agilent Technologies) and hybridized for 17 hours at 60°C in Agilent hybridization chambers in an Agilent hybridization oven rotating at 4 rpm (Agilent Technologies). After hybridization the arrays were washed with an SSPE wash procedure (Agilent Technologies) and scanned with a Scanarray Express HT scanner (Perkin Elmer).

### Data analyses and functional interpretation of microarray data

Spot intensities were quantified using ArrayVision 8.0 (GE Healthcare life sciences). Median density values and background values of each spot were extracted for both the experimental samples (Cy5) and the reference samples (Cy3). Subsequently, quality control was performed for each microarray using both LimmaGUI package in R from Bioconductor and Microsoft Excel. One array in the dietary FOS group did not pass the quality control based on MA plot and signal intensity distribution [72]. Therefore, the dataset contained 23 arrays in total. Data was exported into GeneMaths XT (Applied Maths, Sint-Martens-Latem, Belgium) for background correction and normalization. We discarded spots with an average intensity, over all arrays, of Cy5 lower than 1.5-fold above average background. Then, the Cy5 intensities were normalized against the Cy3 reference as described previously [73]. The data have been deposited in NCBIs Gene Expression Omnibus [74] and are accessible through GEO Series accession number GSE5943. The complete dataset is available in Additional files 4 and 5. Fold changes calculations were performed in Microsoft Excel, fold change equals ratio FOS/control in the case of increase or equals -1/ratio in the case of decrease. For statistical identification of differentially regulated probes between the control and FOS group we used two complementary tests, the often used t-test and Random Forest (RF). T-test was performed in GeneMaths XT, the generated p-values were used to obtain insight into significantly affected genes. To correct for multiple testing we used FDR-adjusted p-values (GeneMaths XT), so called q-values [75]. For t-test we choose a stringent threshold of p < 0.001. The corresponding q-value was 0.035, meaning that 3.5% of the genes selected by this p-value could be false positive. The t-test tests each gene independently and therefore will miss genes that have no main effect but are related to the treatment in gene-gene interaction [76]. We therefore used RF, available as R-package [77,78], as a complementary method as that method includes genes that in gene-gene interaction are related to treatment besides including genes with a main treatment effect. RF was recently successfully used in several microarray studies [19,20]. The method provides an importance index for each gene. This value is dependent on the main treatment effect of a gene. In addition, gene-gene interaction related to the treatment increases importance index value of genes [20]. For RF we defined a threshold where the importance index of each gene in the real dataset exceeded the importance index of genes obtained from analysis of 100 randomly permuted datasets, using randomly assigned class labels FOS or control. This indicates that these genes are truly related to the treatment [79] (detailed method described in [80]). We included the genes that were selected by the t-test threshold and the genes selected by the RF threshold. These genes were considered significantly changed by FOS.

To interpret functional changes in the dataset, we applied two pathway analysis programs, Metacore and GSEA, with different complementary pathway-classification properties. Pathway analysis of the selected genes was performed using MetaCore (GeneGo Inc, St. Joseph, MI). We used classification based on GO-term and classification based on GeneGo annotation. The GeneGo annotation database is a curated database of gene networks based on several databases (KEGG, GO) and scientific literature [21]. We also performed a pathway analysis with GSEA (Broad Institute), a method that does not require preselection of genes by a statistical threshold but uses the whole dataset. GSEA is thoroughly described by Subramanian et al [81]. This method prevents possible selection bias [82,83]. We used the c2 functional genesets based on publicly available and curated databases (GenMapp, Biocarta and SigmaAldrich). Only processes with 5–500 genes were taken into account. Agilent gene annotation version 20060331 was used for both programs. We selected pathways with p < 0.001 in metacore and q-value < 0.25 in GSEA, in accordance with the recommendation of the GSEA developers [81].

Since only about 30% of the genes on the whole genome array were recognized in both pathway programs, we manually supplemented the significantly enriched biological processes with non-annotated genes from the selected gene-set using biological databases (Biocarta, SOURCE, GenMAPP, KEGG) and scientific literature. As processes overlap, we bundled some processes and renamed them.

### Analysis of mRNA expression by Real-time Quantitative RT-PCR

Real-time Quantitative RT-PCR (Q-PCR) was performed on individual samples (n = 12 per group). 1 μg of RNA of all individual samples was used for cDNA synthesis using the iScript cDNA synthesis kit of Bio-Rad Laboratories (Veenendaal, The Netherlands). Real-time reactions were performed by means of the iQ SYBR Green Supermix of Bio-Rad using the MyIQ single-color real-time PCR detection system (Bio-Rad). Each reaction (25 μl) contained 12.5 μl iQ SYBR green supermix, 1 μl forward primer (10 μM), 1 μl reverse primer (10 μM), 8.5 μl RNase-free water and 2 μl diluted cDNA. The following cycles were performed: 1 × 3 min at 95°C, 40 amplification cycles (40 × 10 s 95°C, 45 s 60°C), 1 × 1 min 95°C, 1 × 1 min 62°C and a melting curve (80 × 10 s 55°C with an increase of 0.5°C per 10 s). A negative control without cDNA template was run with every assay. The optimal melting point of dsDNA (Tm) and the efficiency of the reaction were optimized beforehand. Data were normalized against the reference genes Pleckstrin homology domain containing, family A member 6 (*Plekha6*), Nucleoporin 37 (*Nup37*) and β-actin. *Plekha6 *and *Nup37 *were chosen because our microarray data showed equal expression levels for all microarrays, and β-actin was chosen because it is a well accepted reference gene. Primers were designed using Beacon designer 7.00 (Premier Biosoft International, Palo Alto, CA). For primer sequences see Additional file 6. A standard curve for all genes, including reference genes, was generated using serial dilutions of a pooled sample (cDNA from all reactions). mRNA levels were determined using delta CT method (IQ5 software version 2.0, Bio-Rad version). Analysis of all individual samples was performed in duplicate. Data were analyzed using Student's t-test (two-sided) using Prism 4. Differences were considered statistically significant when p < 0.05.

### Protein determination

Mucosal scrapings of individual rats or pools of all rats per group (n = 12) were lysed in a buffer containing 0.125 M TrisHCl pH 6.8, 2% SDS and 20% glycerol. Protein concentrations were determined using DC protein assay kit (Bio-rad Laboratories, Veenendaal, the Netherlands). All samples were boiled in sample buffer (0.125 M TrisHCl pH 6.8; 2% SDS; 20% glycerol; 2% β-mercaptoethanol; 0.04% coomassie briljant blue), and separated by 14% SDS-PAGE. The proteins were transferred onto a nitrocellulose membrane. Immunoblot analysis was performed with a 1:2000 dilution monoclonal antibodies against OXPHOS complexes, Complex IV subunit II (COXII) monoclonal antibody (MS601, Mitosciences, Eugene, OR, USA) or with a 1:200 dilution of monoclonal anti-GLP1 antibody (Abcam (Ab23468), Cambridge, UK) by incubation in 2.5% protifar/TBS-T for 1 1/2 hr at RT. After incubation, blots were washed in TBS-T and incubated 1 hr at RT with a 1:2000 dilution horseradish peroxidase-conjugated anti-mouse IgG (7076, Cell Signaling, Danvers, MA, USA) for detection GLP1 or a 1:2500 dilution of horseradish peroxidase-conjugated anti-mouse IgG (W4021, Promega, Madison, WI, USA) for detection of Complex IV. The signal was detected using an enhanced chemiluminescence detection system (GE Healthcare, The Netherlands) according to the protocol of the supplier. After washing the membranes thoroughly with TBS-T, they were subsequently incubated with the monoclonal anti-Actin (1:100 dilution, Santa Cruz, sc-1615) and HRP anti-goat (1:10000 dilution, Promega. V8051). The intensities of GLP-1, COXII and actin signals on the autoradiography films were quantified using geldoc (Bio-Rad). GLP-1 and COXII quantities were normalized to actin to correct for loading differences.

## Authors' contributions

WR, EK and CV performed the experiments. WR performed the data analysis, interpreted the results and drafted the manuscript. JK and IB participated in the design of the study, in evaluation of the results and in revision of the manuscript. RvdM discussed the results and critically read the manuscript. All authors read and approved the final manuscript.

## Supplementary Material

Additional file 1 Barrier associated genes. The effect of FOS on the expression of potential barrier associated genes. Click here for file

Additional file 2 Functional classification of FOS affected genes by Metacore. Classification of the genes affected by FOS into biological processes analyzed by Metacore. Click here for file

Additional file 3 Genesets enriched in FOS versus control dataset according to GSEA analysis. Classification of the genes affected by FOS into biological processes analyzed by GSEA. Click here for file

Additional file 4 FOS affected genes: Biological processes. Genes affected by FOS and part of a significant biological process. Click here for file

Additional file 5 FOS affected genes: Miscellaneous. Genes affected by FOS but not part of a significantly regulated biological process or genes with unknown function. Click here for file

Additional file 6 Primer sequences. Sequences of the primers used for Q-PCR analysis. Click here for file
