# Supplementary material for: Impaired barrier function by dietary fructo-oligosaccharides (FOS) in rats is accompanied by increased colonic mitochondrial gene expression
Source: BMC Genomics. 2008 Mar 27;9:144. doi: 10.1186/1471-2164-9-144 (PMC2311291; doi:10.1186/1471-2164-9-144)
Supplement: Additional file 1 — Barrier associated genes. The effect of FOS on the expression of potential barrier associated genes. [file 1471-2164-9-144-S1.doc]

**Additional file 1** The effect of FOS on the expression of potential barrier associated genes

| Gene name | Gene name* | Sequence ID | Fold change† | p-value |
| --- | --- | --- | --- | --- |
| **Tight junctions** |  |  |  |  |
| claudin 2 | *Cldn2* | XM_236535 | 1.0 | 0.519 |
| claudin 4 | *Cldn4* | XM_222088 | 1.0 | 0.823 |
| claudin 23 | ***Cldn23*** | XM_224915 | 1.2 | 0.001 |
| Occludin | *Ocln* | NM_031329 | 1.0 | 0.960 |
| cadherin 1 | *Cdh1* | NM_031334 | 1.0 | 0.626 |
| cadherin 5 | *Cdh5* | XM_226213 | 1.0 | 0.924 |
| beta catenin-like 1 | *Ctnlb1* | CB546891 | 1.0 | 0.575 |
| desmocollin 2 | *Dsc2* | XM_226120 | -1.2 | 0.271 |
| junctional adhesion molecule 3 | *Jam3* | NM_001004269 | 1.0 | 0.955 |
| tight junction protein 1 | *Tjp1* | XM_218747 | -1.3 | 0.014 |
| **Apoptosis** |  |  |  |  |
| apoptosis antagonizing transcription factor | ***Aatf*** | NM_053720 | 1.1 | 0.001 |
| apoptosis related protein p18 protein | ***Apr_3*** | XM_216650 | 1.3 | 0.001 |
| apoptotic peptidase activating factor 1 | *Apaf1* | NM_023979 | 1.0 | 0.637 |
| BAX protein, cytoplasmic isoform delta | ***Bax*** | AF235993 | 1.3 | <0.001 |
| B-cell CLL/lymphoma 10 | *Bcl10* | NM_031328 | 1.0 | 0.969 |
| B-cell leukemia/lymphoma 2 | *Bcl2* | NM_016993 | 1.1 | 0.047 |
| BCL2/adenovirus E1B 19kDa-interacting protein 1 | ***Bnip1*** | NM_080897 | 1.2 | 0.001 |
| BCL2-antagonist/killer 1 | ***Bak1*** | NM_053812 | 1.5 | <0.001 |
| Bcl2-associated athanogene 1 | ***Bag1*** | XM_216377 | 1.2 | 0.010 |
| bcl2-associated death promoter | *Bad* | NM_022698 | 1.2 | 0.012 |
| Bcl2-interacting killer | *Biklk* | NM_053704 | 1.1 | 0.299 |
| Bcl2-like 1 | *Bcl2l1* | AW142029 | 1.0 | 0.948 |
| Bcl-2-related ovarian killer protein | *Bok* | NM_017312 | 1.1 | 0.315 |
| BH3 interacting domain death agonist | *Bid* | NM_022684 | 1.0 | 0.793 |
| caspase 2 | *Casp2* | NM_022522 | -1.1 | 0.269 |
| caspase 3, apoptosis related cysteine protease | *Casp3* | NM_012922 | 1.2 | 0.032 |
| caspase 7 | ***Casp7*** | NM_022260 | 1.2 | 0.001 |
| catalase | *Cat* | NM_012520 | 1.3 | 0.014 |
| CDC42 effector protein (Rho GTPase binding) 5 | ***Cdc42ep5*** | XM_341784 | 1.2 | 0.016 |
| CDC42 small effector 1 | ***Cdc42se1*** | AW920756 | 1.2 | 0.001 |
| cell division cycle 42 homolog (S. cerevisiae) | ***Cdc42*** | NM_171994 | 1.3 | 0.006 |
| cell division cycle and apoptosis regulator 1 | ***Ccar1*** | XM_342143 | 1.2 | 0.001 |
| deoxyribonuclease I | ***Dnase1*** | NM_013097 | 2.3 | <0.001 |
| programmed cell death 6 | ***Pdcd6*** | XM_217732 | 1.2 | <0.001 |
| programmed cell death 8 | ***Pdcd8*** | NM_031356 | 1.3 | 0.001 |
| PYD and CARD domain containing | ***Pycard*** | NM_172322 | 1.6 | <0.001 |
| STEAP family member 3 | ***Steap3*** | NM_133314 | 1.4 | <0.001 |
| TatD DNase domain containing 1 | ***Tatdn1*** | XM_228158 | 1.3 | <0.001 |
| transmembrane BAX inhibitor motif containing 4 | ***Tmbim4*** | NM_199116 | 1.2 | 0.001 |
| v-crk sarcoma virus CT10 oncogene homolog (avian) | ***Crk*** | BG671506 | 1.1 | 0.013 |
| **Mucosal defense** |  |  |  |  |
| mucin 2 | *Muc2* | U07615 | -1.2 | 0.381 |
| mucin 3 | *Muc3* | U76551 | 1.1 | 0.519 |
| gastric mucin | ***Muc*** | XM_344685 | 1.7 | <0.001 |
| defensin NP-4 precursor | *Np4* | NM_173299 | -1.4 | 0.026 |
| defensin related cryptdin 4 | *RD-5* | XM_214386 | -1.1 | 0.730 |
| defensin, alpha 5, Paneth cell-specific | *Defa* | NM_173329 | -1.1 | 0.090 |
| lipocalin 2 | *Lcn2* | NM_130741 | 1.0 | 0.635 |
| phospholipase A2, group IIA (platelets, synovial fluid) | ***Pla2g2a*** | NM_031598 | 3.7 | <0.001 |
| matrix metallopeptidase 7 | *Mmp7* | NM_012864 | -1.1 | 0.111 |
| lipopolysaccharide binding protein | *Lbp* | NM_017208 | 1.7 | 0.018 |
| cathelicidin antimicrobial peptide | *CRAMP* | CA509601 | 1.1 | 0.173 |
| S100 calcium binding protein A8 (calgranulin A) | *S100a8* | NM_053822 | 1.3 | 0.091 |
| S100 calcium binding protein A9 (calgranulin B) | *S100a9* | NM_053587 | 1.4 | 0.003 |
| toll-like receptor 1 | ***Tlr1*** | XM_223421 | 1.5 | <0.001 |
| toll-like receptor 2 | *Tlr2* | NM_198769 | 1.1 | 0.294 |
| toll-like receptor 3 | *Tlr3* | NM_198791 | 1.1 | 0.489 |
| toll-like receptor 4 | *Tlr4* | NM_019178 | -1.1 | 0.150 |
| toll-like receptor 5 | *Tlr5* | XM_223016 | 1.1 | 0.588 |
| toll-like receptor 9 | *Tlr9* | NM_198131 | 1.0 | 0.732 |
| trefoil factor 1 | ***Tff1*** | NM_057129 | 1.6 | <0.001 |
| trefoil factor 3 | ***Tff3*** | NM_013042 | 1.7 | <0.001 |
| beta-2 microglobulin | ***B2m*** | NM_012512 | 1.4 | <0.001 |
| MHC class II region expressed gene KE2 | ***Ke2*** | NM_212506 | 1.4 | <0.001 |
| matrix metallopeptidase 7 | *Mmp7* | NM_012864 | -1.1 | 0.111 |
| lipopolysaccharide binding protein | *Lbp* | NM_017208 | 1.7 | 0.018 |
| immunoglobulin joining chain | *Igj* | XM_341195 | 1.2 | 0.422 |
| CD79A antigen (immunoglobulin-associated alpha) | *Iga* | XM_001077003 | 1.0 | 0.987 |
| lectin, mannose-binding 2 | ***Lman2*** | XM_214428 | 1.3 | 0.001 |

* Genes significantly regulated by FOS are shown in bold gene symbols.

†Ratio FOS diet/ Control diet.
