# Supplementary material for: Impaired barrier function by dietary fructo-oligosaccharides (FOS) in rats is accompanied by increased colonic mitochondrial gene expression
Source: BMC Genomics. 2008 Mar 27;9:144. doi: 10.1186/1471-2164-9-144 (PMC2311291; doi:10.1186/1471-2164-9-144)
Supplement: Additional file 2 — Functional classification of FOS affected genes by Metacore. Classification of the genes affected by FOS into biological processes analyzed by Metacore. [file 1471-2164-9-144-S2.doc]

**Additional file 2** Functional classification of FOS affected genes by Metacore.

| **Process*** | **Number of genes** | | **p-value**^§^ |
| --- | --- | --- | --- |
|  | **Selection** | **Total** |  |
| Generation of precursor metabolites and energy ^1^ | 53 | 323 | 2.3E-17 |
| Translation: Translation in mitochondria ^2^ | 31 | 187 | 6.7E-13 |
| Mitochondrial electron transport, NADH to ubiquinone ^1^ | 11 | 30 | 2.6E-08 |
| Protein targeting to mitochondrion ^1^ | 10 | 25 | 4.3E-08 |
| Mitochondrial transport ^1^ | 12 | 41 | 1.1E-07 |
| Electron transport ^1^ | 19 | 108 | 1.9E-07 |
| Oxidative phosphorylation ^1^ | 13 | 52 | 2.5E-07 |
| ATP synthesis coupled electron transport ^1^ | 11 | 37 | 3.1E-07 |
| Proteolysis: Ubiquitin-proteasomal proteolysis ^2^ | 21 | 167 | 6.1E-07 |
| Response to hypoxia and oxidative stress ^2^ | 13 | 93 | 2.9E-05 |
| Coenzyme metabolic process ^1^ | 14 | 91 | 3.8E-05 |
| Regulation of carbohydrate metabolic process ^1^ | 6 | 17 | 5.7E-05 |
| Protein thiol-disulfide exchange ^1^ | 3 | 3 | 8.4E-05 |
| Immune: antigen presentation ^2^ | 19 | 196 | 9.1E-05 |
| Carbohydrate metabolic process ^1^ | 30 | 328 | 1.1E-04 |
| Muscle filament sliding ^1^ | 4 | 8 | 2.2E-04 |
| Energy derivation by oxidation of organic compounds ^1^ | 16 | 133 | 2.3E-04 |
| Cytoskeleton-dependent intracellular transport ^1^ | 11 | 73 | 3.1E-04 |
| Cofactor metabolic process ^1^ | 15 | 125 | 3.6E-04 |
| Response to copper ion ^1^ | 4 | 9 | 3.9E-04 |
| Response to inorganic substance ^1^ | 7 | 34 | 5.7E-04 |
| Main pathways of carbohydrate metabolic process ^1^ | 10 | 68 | 7.1E-04 |
| Nucleosome assembly ^1^ | 7 | 36 | 8.3E-04 |

* Biological processes based on ^1^Metacore GO-analysis or ^2^Metacore GeneGo-analysis.

† The number of genes affected by FOS (selection) and the total number of genes present in the process are given (total).

§ Metacore derived p-value.

Processes containing >500 genes were excluded from the analysis, as these processes represent uninformative broad classes such as “biological function”.
