# Supplementary material for: Impaired barrier function by dietary fructo-oligosaccharides (FOS) in rats is accompanied by increased colonic mitochondrial gene expression
Source: BMC Genomics. 2008 Mar 27;9:144. doi: 10.1186/1471-2164-9-144 (PMC2311291; doi:10.1186/1471-2164-9-144)
Supplement: Additional file 3 — Genesets enriched in FOS versus control dataset according to GSEA analysis. Classification of the genes affected by FOS into biological processes analyzed by GSEA. [file 1471-2164-9-144-S3.doc]

**Additional file 3** Genesets enriched in FOS versus control dataset according to GSEA analysis.

| **Process *** | **N** | **ES** | **NES** | **q-value**† |
| --- | --- | --- | --- | --- |
| Oxidative phosphorylation^1^ | 25 | 0.65 | 1.96 | 0.07 |
| Propanoate metabolism^1^ | 24 | 0.62 | 1.92 | 0.10 |
| Proteasome^1^ | 16 | 0.82 | 1.83 | 0.16 |
| Free Radical Induced Apoptosis^2^ | 8 | 0.72 | 1.80 | 0.19 |
| Butanoate metabolism^1^ | 20 | 0.66 | 1.79 | 0.19 |
| Programmed cell death^3^ | 10 | 0.68 | 1.77 | 0.20 |
| Valine, leucine and isoleucine degradation^1^ | 27 | 0.62 | 1.75 | 0.23 |
| Krebs TCA cycle^1^ | 22 | 0.63 | 1.74 | 0.25 |
| Proteasome pathway^2^ | 17 | 0.77 | 1.73 | 0.25 |

* Biological processes based on ^1^GenMapp, ^2^Biocart and ^3^SigmaAldrich.

† Q-value>0.25 was used as cut-off as advised by GSEA

N, Number of genes in the geneset. ES, enrichment score for the gene set. NES, normalized enrichment score. FDR derived q-values.
