# Supplementary material for: Impaired barrier function by dietary fructo-oligosaccharides (FOS) in rats is accompanied by increased colonic mitochondrial gene expression
Source: BMC Genomics. 2008 Mar 27;9:144. doi: 10.1186/1471-2164-9-144 (PMC2311291; doi:10.1186/1471-2164-9-144)
Supplement: Additional file 4 — FOS affected genes: Biological processes. Genes affected by FOS and part of a significant biological process. [file 1471-2164-9-144-S4.doc]

**Additional file 4: Biological processes**

Genes affected by FOS and part of a significantly regulated biological process.

Genes included in this selection have a p-values<0.001 or are selected by RF.

| **Biological process** | **Gene name** | **Gene symbol** | **Sequence ID** | **Fold change** | **P-value** |
| --- | --- | --- | --- | --- | --- |
| **Mitochondria** | |  |  |  |  |
| ***Complex I*** | |  |  |  |  |
|  | NADH dehydrogenase (ubiquinone) 1 beta subcomplex, 6 | *Ndufb6* | XM_216378 | 1.54 | <0.001 |
|  | NADH dehydrogenase (ubiquinone) Fe-S protein 5b, 15kDa (NADH-coenzyme Q reductase) | *Ndufs5a* | XM_227473 | 1.54 | <0.001 |
|  | NADH dehydrogenase (ubiquinone) Fe-S protein 6 | *Ndufs6* | NM_019223 | 1.48 | <0.001 |
|  | NADH dehydrogenase (ubiquinone) 1 beta subcomplex, 9 | *Ndufb9* | XM_216929 | 1.47 | <0.001 |
|  | NADH dehydrogenase (ubiquinone) flavoprotein 2 | *Ndufv2* | NM_031064 | 1.47 | <0.001 |
|  | NADH-ubiquinone oxidoreductase B9 subunit (Complex I-B9) (CI-B9) | *Ndufa3* | XM_341773 | 1.46 | <0.001 |
|  | NADH dehydrogenase (ubiquinone) 1 beta subcomplex 3 | *Ndufb3* | XM_344609 | 1.46 | <0.001 |
|  | NADH dehydrogenase (ubiquinone) 1 alpha subcomplex, 2 | *Ndufa2* | XM_214570 | 1.46 | <0.001 |
|  | NADH dehydrogenase (ubiquinone) Fe-S protein 8 | *Ndufs8* | XM_215197 | 1.45 | <0.001 |
|  | NADH dehydrogenase (ubiquinone) 1 beta subcomplex 8 | *Ndufb8* | XM_215269 | 1.45 | <0.001 |
|  | NADH dehydrogenase (ubiquinone) flavoprotein 3-like | *Nduf3* | NM_022607 | 1.44 | <0.001 |
|  | NADH-ubiquinone oxidoreductase PDSW subunit (Complex I-PDSW) | *Ndufb10* | XM_213242 | 1.42 | <0.001 |
|  | NADH dehydrogenase (ubiquinone) 1NADH dehydrogenase (ubiquinone) 1alpha subcomplex, 13 | *Ndufa13* | XR_008769 | 1.41 | 0.001 |
|  | NADH dehydrogenase (ubiquinone) 1 beta subcomplex, 7 | *Ndufb7* | XM_341664 | 1.40 | 0.001 |
|  | NADH dehydrogenase (ubiquinone) 1, alpha/beta subcomplex, 1 | *Ndufab1* | XM_215044 | 1.38 | <0.001 |
|  | NADH dehydrogenase (ubiquinone) 1 alpha subcomplex, 12 | *Ndufa12* | XM_216880 | 1.38 | <0.001 |
|  | NADH dehydrogenase (ubiquinone) 1 beta subcomplex, 11 | *Ndufb11* | XM_216785 | 1.37 | <0.001 |
|  | NADH dehydrogenase (ubiquinone) 1 alpha subcomplex, 4 | *Ndufa4* | NM_010886 | 1.36 | <0.001 |
|  | NADH dehydrogenase (ubiquinone) 1 beta subcomplex, 5 | *Ndufb5* | XM_215544 | 1.34 | <0.001 |
|  | NADH dehydrogenase (ubiquinone) 1 alpha subcomplex 11 | *Ndufa11* | NM_212517 | 1.33 | <0.001 |
|  | NADH dehydrogenase (ubiquinone) 1, subcomplex unknown, 2 | *Ndufc2* | NM_001009290 | 1.32 | <0.001 |
|  | NADH dehydrogenase (ubiquinone) 1 alpha subcomplex, 8 | *Ndufa8* | XM_216044 | 1.32 | <0.001 |
|  | NADH dehydrogenase 1 alpha subcomplex 10-like protein | *Ndufa10l* | NM_182671 | 1.30 | <0.001 |
|  | NADH dehydrogenase (ubiquinone) 1 alpha subcomplex, 7 (B14.5a) | *Ndufa7* | XM_216859 | 1.28 | 0.002 |
|  | glycerol-3-phosphate dehydrogenase 2, mitochondrial | *Gpd2* | NM_012736 | 1.27 | 0.001 |
|  | NADH dehydrogenase (ubiquinone) Fe-S protein 3 | *Ndufs3* | XM_215776 | 1.26 | <0.001 |
|  | NADH dehydrogenase (ubiquinone) flavoprotein 1 | *Ndufv1* | NM_001006972 | 1.24 | <0.001 |
| ***Complex II*** | |  |  |  |  |
|  | electron-transferring-flavoprotein dehydrogenase | *Etfdh* | NM_198742 | 1.51 | <0.001 |
|  | succinate dehydrogenase complex, subunit B, iron sulfur (Ip) | *Sdhb* | XM_216558 | 1.43 | <0.001 |
|  | succinate-CoA ligase, GDP-forming, alpha subunit | *Suclg1* | NM_053752 | 1.36 | <0.001 |
|  | Succinate dehydrogenase A | *Sdha* | XM_215279 | 1.35 | <0.001 |
|  | succinate dehydrogenase complex, subunit C, integral membrane protein | *Sdhc* | NM_001005534 | 1.31 | <0.001 |
| ***Complex III*** | |  |  |  |  |
|  | ubiquinol-cytochrome c reductase binding protein | *Uqcrb* | XM_224385 | 1.53 | <0.001 |
|  | ubiquinol-cytochrome c reductase hinge protein | *Uqcrh* | NM_001009480 | 1.39 | <0.001 |
|  | ubiquinol-cytochrome c reductase complex 7.2kDa protein isoform a | *Uqcrc* | BF556101 | 1.36 | <0.001 |
| ***Complex IV*** | |  |  |  |  |
|  | cytochrome c oxidase subunit VIIb | *Cox7b* | NM_182819 | 1.61 | <0.001 |
|  | cytochrome c oxidase, subunit VIc | *Cox6c* | NM_019360 | 1.54 | <0.001 |
|  | cytochrome c oxidase subunit Vb | *Cox5b* | NM_053586 | 1.51 | <0.001 |
|  | cytochrome c oxidase, subunit VIIa 2 | *Cox7a3* | NM_022503 | 1.50 | <0.001 |
|  | cytochrome c oxidase subunit VIIa-L precursor | *LOC365879* | XM_345256 | 1.50 | <0.001 |
|  | cytochrome c oxidase subunit VIc-1 | *COX-VIc-1* | NM_173303 | 1.50 | <0.001 |
|  | cytochrome c oxidase, subunit VIa, polypeptide 1 | *Cox6a1* | XM_341094 | 1.48 | <0.001 |
|  | Cytochrome c, somatic | *Cytc* | XM_218990 | 1.34 | 0.001 |
|  | cytochrome c oxidase, subunit Va | *Cox5a* | NM_145783 | 1.30 | <0.001 |
|  | cytochrome c oxidase, subunit VIIIa | *Cox8a* | L48209 | 1.29 | <0.001 |
|  | SCO cytochrome oxidase deficient homolog 1 (yeast) |  | XM_573117 | 1.28 | 0.001 |
|  | COX10 homolog, cytochrome c oxidase assembly protein, heme A: farnesyltransferase | *Cox10* | TC475388 | 1.24 | <0.001 |
|  | COX4 neighbor | *Cox4* | XM_341703 | 1.20 | 0.002 |
| ***Complex V*** | |  |  |  |  |
|  | ATPase inhibitory factor 1 | *Atpi* | NM_012915 | 1.59 | <0.001 |
|  | ATPase inhibitory factor 1 | *If1* | TC512274 | 1.54 | <0.001 |
|  | ATP synthase, H+ transporting, mitochondrial F0 complex, subunit f, isoform 2 | *Atpf2* | BM986304 | 1.52 | <0.001 |
|  | ATP synthase, H+ transporting, mitochondrial F0 complex, subunit G | *Atp5g* | XM_345982 | 1.52 | <0.001 |
|  | ATP synthase, H+ transporting, mitochondrial F0 complex, subunit e | *Atp5i* | NM_080481 | 1.49 | <0.001 |
|  | ATP synthase D chain, mitochondrial | *Atpd* | XM_224879 | 1.48 | <0.001 |
|  | ATP synthase, H+ transporting, mitochondrial F0 complex, subunit d | *Atp5h* | NM_019383 | 1.46 | <0.001 |
|  | ATP synthase, H+ transporting, mitochondrial F0 complex, subunit F6 | *Atp5j* | NM_053602 | 1.43 | <0.001 |
|  | ATP synthase, H+ transporting, mitochondrial F0 complex, subunit b, isoform 1 | *Atp5f1* | NM_134365 | 1.37 | <0.001 |
|  | ATP synthase, H+ transporting, mitochondrial F1 complex, O subunit | *Atp5o* | NM_138883 | 1.36 | <0.001 |
|  | ATP synthase lipid-binding protein, mitochondrial precursor (ATP synthase proteolipid P1) | *Atpc* | XM_222276 | 1.32 | <0.001 |
|  | ATP binding domain 3 | *Atpb3* | XM_218640 | 1.30 | <0.001 |
|  | ATP-binding cassette, sub-family G (WHITE), member 4 | *Abcg4* | XM_236186 | 1.29 | <0.001 |
|  | ATP synthase, H+ transporting, mitochondrial F1 complex, delta subunit | *Atp5d* | NM_139106 | 1.28 | <0.001 |
|  | ATP synthase, H+ transporting, mitochondrial F0 complex, subunit c (subunit 9), isoform 1 | *Atp5g1* | NM_017311 | 1.25 | <0.001 |
|  | ATPase, H transporting, lysosomal V1 subunit G1 | *Atpv1g* | XM_216411 | 1.25 | <0.001 |
|  | ATP-binding cassette, sub-family A (ABC1), member 2 | *Abca2* | NM_024396 | 1.25 | 0.007 |
|  | ATP synthase, H+ transporting, mitochondrial F0 complex, subunit c (subunit 9), isoform 2 | *Atp5g2* | NM_133556 | 1.21 | <0.001 |
|  | ATPase, H+ transporting, V1 subunit A, isoform 1 | *Atpv1* | TC465908 | 1.17 | 0.001 |
|  | ATPase, Na+/K+ transporting, beta 1 polypeptide | *Atp1b1* | NM_013113 | 1.16 | 0.001 |
|  | ATP-binding cassette, sub-family A (ABC1), member 1 | *Abca1* | NM_178095 | 1.15 | 0.009 |
| ***Metabolism and TCA cycle*** | |  |  |  |  |
|  | aldo-keto reductase family 1, member B8 | *Akr1b8* | NM_173136 | 2.13 | <0.001 |
|  | aldo-keto reductase family 1, member C12 | *Akr1c12* | XM_341549 | 1.88 | <0.001 |
|  | 3-hydroxybutyrate dehydrogenase (heart, mitochondrial) | *Bdh* | AW918701 | 1.85 | <0.001 |
|  | quinolinate phosphoribosyltransferase | *Qprt* | NM_001009646 | 1.82 | <0.001 |
|  | diazepam binding inhibitor | *Dbi* | NM_031853 | 1.68 | <0.001 |
|  | pseudogene for diazepam binding inhibitor 1 | *Dbii1* | XM_237195 | 1.67 | <0.001 |
|  | malic enzyme 1 | *Me1* | M30596 | 1.65 | <0.001 |
|  | aldehyde dehydrogenase 2 | *Aldh2* | NM_032416 | 1.63 | <0.001 |
|  | acetyl-Coenzyme A acyltransferase 2 (mitochondrial 3-oxoacyl-Coenzyme A thiolase) | *Acaa2* | NM_130433 | 1.56 | <0.001 |
|  | Propionyl-CoA carboxylase alpha chain, mitochondrial precursor (PCCase alpha subunit) | *Pcca* | XM_341383 | 1.48 | <0.001 |
|  | creatine kinase, brain | *Ckb* | NM_012529 | 1.48 | <0.001 |
|  | demethyl-Q 7 | *Coq7* | NM_012785 | 1.44 | <0.001 |
|  | acyl-Coenzyme A dehydrogenase, very long chain | *Acadvl* | NM_012891 | 1.43 | <0.001 |
|  | hydroxyacyl-Coenzyme A dehydrogenase type II | *Hsd17b10* | NM_031682 | 1.41 | <0.001 |
|  | sterol carrier protein 2 | *Scp2* | NM_138508 | 1.39 | <0.001 |
|  | malic enzyme 2, NAD(+)-dependent, mitochondrial | *Me2* | XM_225729 | 1.38 | <0.001 |
|  | 3-hydroxy-3-methylglutaryl-Coenzyme A synthase 1 | *Hmgcs1* | NM_017268 | 1.38 | 0.008 |
|  | lactamase, beta 2 | *Lactb2* | XM_216316 | 1.38 | <0.001 |
|  | malate dehydrogenase 1, NAD (soluble) | *Mdh1* | NM_033235 | 1.38 | 0.002 |
|  | glyceraldehyde-3-phosphate dehydrogenase | *Gapdh* | XM_233263 | 1.36 | <0.001 |
|  | enoyl Coenzyme A hydratase domain containing 1 | *Echdc1* | NM_001007734 | 1.35 | <0.001 |
|  | fructose-1,6- biphosphatase 1 | Fbp1 | NM_012558 | 1.35 | 0.001 |
|  | hydroxyacyl-Coenzyme A dehydrogenase/3-ketoacyl-Coenzyme A thiolase/ alpha subunit | *Hadha* | NM_130826 | 1.35 | <0.001 |
|  | fumarate hydratase 1 | *Fh1* | NM_017005 | 1.35 | <0.001 |
|  | degenerative spermatocyte homolog 2 (Drosophila), lipid desaturase | *Degs2* | XM_234532 | 1.34 | <0.001 |
|  | nudix (nucleoside diphosphate linked moiety X)-type motif 19 | *Nudt19* | NM_001004258 | 1.34 | <0.001 |
|  | acyl-CoA synthetase short-chain family member 1 | *Acas2l* | XM_215897 | 1.32 | <0.001 |
|  | isocitrate dehydrogenase 3 (NAD+) beta | *Idh3B* | NM_053581 | 1.32 | <0.001 |
|  | mitochondrial acyl-CoA thioesterase 1 | *Mte1* | NM_138907 | 1.32 | <0.001 |
|  | aldo-keto reductase family 1, member B4 (aldose reductase) | *Aldr1* | NM_012498 | 1.31 | 0.001 |
|  | gamma-glutamyl hydrolase | Ggh | NM_012960 | 1.31 | 0.001 |
|  | dihydrolipoamide S-succinyltransferase (E2 component of 2-oxo-glutarate complex) | *Dlst* | NM_001006981 | 1.31 | <0.001 |
|  | Fumarate hydratase, mitochondrial precursor (Fumarase) | *Fh* | XR_008238 | 1.30 | <0.001 |
|  | acetyl-coenzyme A carboxylase alpha | *Acaca* | X53003 | 1.30 | <0.001 |
|  | glutaredoxin 5 homolog (S. cerevisiae) | *Glrx5* | XM_343103 | 1.30 | <0.001 |
|  | aldo-keto reductase family 1, member E1 | *Akr1e1* | NM_001008342 | 1.30 | 0.001 |
|  | glutaredoxin 2 (thioltransferase) | *Glrx2* | XM_213890 | 1.28 | 0.002 |
|  | fumarate hydratase 1 | *Fh1* | NM_017005 | 1.27 | <0.001 |
|  | solute carrier family 25 (mitochondrial carrier; adenine nucleotide translocator), member 3 | *ANT* | NM_139100 | 1.26 | <0.001 |
|  | ACN9 homolog (S. cerevisiae) | *Acn9* | XM_342641 | 1.25 | <0.001 |
|  | dihydrolipoamide dehydrogenase | *Dld* | NM_199385 | 1.24 | <0.001 |
|  | carbonic anhydrase 5a, mitochondrial | *Ca5a* | NM_019293 | 1.24 | <0.001 |
|  | Aldose reductase (AR) (Aldehyde reductase) | *Ar* | XM_226245 | 1.23 | 0.001 |
|  | Nonspecific lipid-transfer protein, mitochondrial precursor | *Nsldp* | XM_345499 | 1.23 | <0.001 |
|  | enoyl Coenzyme A hydratase, short chain, 1, mitochondrial | *Echs1* | NM_078623 | 1.23 | 0.001 |
|  | exocyst complex component 3 | *Exoc3* | XM_341745 | 1.23 | 0.001 |
|  | retinoblastoma binding protein 6 | *Rbbp6* | XM_219296 | 1.22 | <0.001 |
|  | pyruvate dehydrogenase kinase, isoenzyme 3 | *Pdk3* | LOC363473 | 1.22 | 0.001 |
|  | 3-hydroxy-3-methylglutaryl-Coenzyme A lyase | *Hmgcl* | NM_024386 | 1.18 | 0.001 |
|  | diacylglycerol O-acyltransferase 1 | *Dgat1* | NM_053437 | 1.15 | 0.001 |
|  | novel Acyl Transferase C. elegans C50D2.7 (variant 1) | *At1* | XM_235527 | 1.13 | 0.010 |
|  | glucose phosphate isomerase | *Gpi* | BQ193604 | -1.28 | <0.001 |
|  | 3-oxoacid CoA transferase 2A | *Oxct2a* | XM_233493 | -1.23 | 0.001 |
|  | adenosine monophosphate deaminase 2 (isoform L) | *Ampd2* | XM_342313 | -1.20 | 0.008 |
| ***Mitochondrial ribosomes*** | |  |  |  |  |
|  | mitochondrial ribosomal protein S16 | *Mrps16* | XM_214132 | 1.43 | <0.001 |
|  | mitochondrial ribosomal protein L22 | *Mrpl22* | XM_213307 | 1.42 | <0.001 |
|  | mitochondrial ribosomal protein L41 | *Mrpl41* | XM_216010 | 1.39 | <0.001 |
|  | mitochondrial ribosomal protein L40 | *Mrpl40* | XM_213588 | 1.38 | <0.001 |
|  | mitochondrial ribosomal protein L13 | *Mrpl13* | XM_236903 | 1.38 | <0.001 |
|  | mitochondrial ribosomal protein S36 | *Mrps36* | XM_215468 | 1.37 | <0.001 |
|  | mitochondrial ribosomal protein S25 | *Mrps25* | XM_216222 | 1.37 | <0.001 |
|  | mitochondrial ribosomal protein L35 | *Mrpl35* | XM_216169 | 1.37 | <0.001 |
|  | ribosomal protein, mitochondrial, L12 | *Mrpl12* | XM_221202 | 1.35 | <0.001 |
|  | mitochondrial ribosomal protein L11 | *mrpl11* | NM_001006973 | 1.34 | <0.001 |
|  | mitochondrial ribosomal protein L42 isoform b | *Mrlp42b* | XM_213981 | 1.33 | 0.001 |
|  | mitochondrial ribosomal protein L42 | *Mrpl42* | XM_216882 | 1.33 | 0.001 |
|  | mitochondrial ribosomal protein L24 | *mrpl24* | NM_001007637 | 1.33 | <0.001 |
|  | mitochondrial ribosomal protein L47 | *Mrpl47* | XM_215546 | 1.32 | <0.001 |
|  | mitochondrial ribosomal protein S14 | *Mrps14* | XM_213906 | 1.32 | <0.001 |
|  | mitochondrial ribosomal protein L20 | *Mrpl20* | XM_216599 | 1.32 | <0.001 |
|  | mitochondrial ribosomal protein S15 | *Mrps15* | NM_001007653 | 1.30 | <0.001 |
|  | mitochondrial ribosomal protein L18 | *Mrpl18* | XM_214751 | 1.30 | 0.001 |
|  | mitochondrial ribosomal protein L50 | *Mrpl50* | XM_342835 | 1.30 | <0.001 |
|  | mitochondrial ribosomal protein L51 | *Mrpl51* | XM_216269 | 1.29 | <0.001 |
|  | mitochondrial ribosomal protein L53 | *Mrpl53* | XM_342712 | 1.28 | 0.005 |
|  | mitochondrial ribosomal protein L32 | *Mrpl32* | XM_214491 | 1.27 | <0.001 |
|  | mitochondrial ribosomal protein L2 | *Mrpl2* | XM_217355 | 1.27 | <0.001 |
|  | mitochondrial ribosomal protein S18A | *Mrps18a* | NM_198756 | 1.27 | <0.001 |
|  | mitochondrial ribosomal protein S23 | *Mrps23* | XM_340874 | 1.27 | <0.001 |
|  | mitochondrial ribosomal protein L48 | *Mrpl48* | XM_215009 | 1.27 | 0.001 |
|  | mitochondrial ribosomal protein L55 | *Mrpl55* | XM_213321 | 1.26 | 0.001 |
|  | mitochondrial ribosomal protein L1 | *Mrpl1* | XM_214008 | 1.25 | 0.001 |
|  | mitochondrial ribosomal protein L16 | *Mrpl16* | NM_001009647 | 1.25 | <0.001 |
|  | mitochondrial ribosomal protein L44 | *Mrpl44* | XM_217450 | 1.24 | 0.002 |
|  | mitochondrial ribosomal protein S12 | *Mrps1* | XM_214890 | 1.23 | <0.001 |
|  | mitochondrial ribosomal protein S26 | *Mrps26* | XM_342520 | 1.20 | 0.002 |
|  | mitochondrial ribosomal protein S5 | *Mrps5* | XM_215833 | 1.19 | <0.001 |
| ***Protein transport*** | |  |  |  |  |
|  | translocase of inner mitochondrial membrane 8 homolog b (yeast) | *Timm8b* | NM_022541 | 1.45 | <0.001 |
|  | translocase of inner mitochondrial membrane 13 homolog (yeast) | *Timm13* | NM_145781 | 1.35 | <0.001 |
|  | translocase of the inner mitochondrial membrane 14 isoform a | *Timm14a* | TC475929 | 1.34 | <0.001 |
|  | translocase of inner mitochondrial membrane 10 homolog (yeast) | *Timm10* | NM_172074 | 1.31 | 0.001 |
|  | inner membrane protein, mitochondrial | *Immt* | XM_232055 | 1.30 | <0.001 |
|  | mitochondrial intermediate peptidase | *Mipep* | NM_031052 | 1.30 | <0.001 |
|  | sorting and assembly machinery component 50 homolog (S. cerevisiae) | *Samm50* | NM_001004241 | 1.27 | <0.001 |
|  | translocase of inner mitochondrial membrane 17 homolog B (yeast) | *Tim17b* | XM_228758 | 1.25 | <0.001 |
|  | translocase of inner mitochondrial membrane 23 homolog (yeast) | *Timm23* | NM_019352 | 1.24 | <0.001 |
|  | translocator of inner mitochondrial membrane 17a | *Timm17a* | NM_019351 | 1.19 | 0.002 |
|  | translocator of inner mitochondrial membrane 44 | *Timm44* | NM_017267 | 1.18 | 0.001 |
| ***Miscellaneous*** | |  |  |  |  |
|  | carnitine palmitoyltransferase 2 | *Cpt2* | NM_012930 | 1.47 | <0.001 |
|  | 6.8 kDa mitochondrial proteolipid |  | AW917139 | 1.45 | <0.001 |
|  | coiled-coil-helix-coiled-coil-helix domain containing 2 | *Chchd2* | XM_237424 | 1.43 | <0.001 |
|  | cytochrome b-245, alpha polypeptide | Cyba | NM_024160 | 1.42 | <0.001 |
|  | upregulated during skeletal muscle growth 5 | *Usmg5* | NM_133544 | 1.41 | <0.001 |
|  | heat-responsive protein 12 | *Hrsp12* | NM_031714 | 1.39 | <0.001 |
|  | solute carrier family 25 (mitochondrial carnitine/acylcarnitine translocase), member 20 | *Slc25a20* | NM_053965 | 1.35 | 0.002 |
|  | coiled-coil-helix-coiled-coil-helix domain containing 1 | *Chchd1* | XM_341280 | 1.33 | 0.001 |
|  | prohibitin 2 | *Bcap37* | XM_342755 | 1.26 | 0.001 |
|  | prohibitin | *Phb* | NM_031851 | 1.24 | <0.001 |
|  | protein tyrosine phosphatase, mitochondrial 1 | *Plip* | XM_342460 | 1.22 | <0.001 |
|  | coiled-coil-helix-coiled-coil-helix domain containing 3 | *Chchd3* | XM_238346 | 1.16 | 0.001 |
|  |  |  |  |  |  |
| **Protein turnover** | |  |  |  |  |
| ***Protein degradation*** | |  |  |  |  |
|  | proteasome (prosome, macropain) 28 subunit, beta | *Psme2* | NM_017257 | 1.48 | <0.001 |
|  | proteasome subunit alpha type 3-like | *Psma3l* | BN000326 | 1.39 | <0.001 |
|  | Ubiquitin-Like 5 Protein | *Ubl5* | AW919138 | 1.38 | <0.001 |
|  | proteasome (prosome, macropain) subunit, alpha type 3 | *Psma3* | NM_017280 | 1.36 | <0.001 |
|  | proteasome (prosome, macropain) subunit, alpha type 7 | *Psma7* | NM_001008217 | 1.35 | <0.001 |
|  | proteasome (prosome, macropain) subunit, alpha type 2 | *Psma2* | NM_017279 | 1.35 | <0.001 |
|  | Proteasome activator complex subunit 1 (Proteasome activator 28-alpha subunit) | *Pa23a* | XM_344615 | 1.34 | 0.001 |
|  | proteasome (prosome, macropain) subunit, beta type 6 | *Psmb6* | XM_345013 | 1.31 | <0.001 |
|  | DNA-damage inducible protein 1 | *Ddi1* | XM_345895 | 1.31 | <0.001 |
|  | peptidase (mitochondrial processing) beta | *Pmpcb* | NM_022395 | 1.30 | <0.001 |
|  | ubiquitin A-52 residue ribosomal protein fusion product 1 | *Uba52* | NM_031687 | 1.29 | 0.001 |
|  | proteasome subunit R-IOTA | *Psma6* | AW141337 | 1.27 | <0.001 |
|  | proteasome (prosome, macropain) 26S subunit, non-ATPase, 12 | *Psmd12* | NM_001005875 | 1.27 | <0.001 |
|  | ubiquitin-conjugating enzyme E2A, RAD6 homolog (S. cerevisiae) | *Ube2a* | XM_216466 | 1.27 | <0.001 |
|  | proteasome (prosome, macropain) subunit, beta type 3 | *Psmb3* | NM_017285 | 1.26 | <0.001 |
|  | proteasome (prosome, macropain) subunit, beta type 4 | *Psmb4* | NM_031629 | 1.26 | <0.001 |
|  | Proteasome subunit beta type 3 (Proteasome theta chain) | *Psmb3* | XR_007494 | 1.26 | <0.001 |
|  | Ubiquitin carboxyl-terminal hydrolase isozyme L3 (Ubiquitin thiolesterase L3) | *Uchl3* | XM_345541 | 1.25 | <0.001 |
|  | proteasome (prosome, macropain) 26S subunit, ATPase, 6 | *Psmc6* | XM_214147 | 1.24 | <0.001 |
|  | ubiquitin-conjugating enzyme E2S | *Ube2s* | XM_214806 | 1.23 | <0.001 |
|  | proteasome (prosome, macropain) 26S subunit, non-ATPase, 1 | *Psmd1* | NM_031978 | 1.22 | <0.001 |
|  | peptidase (prosome, macropain) 26S subunit, ATPase 1 | *Psmc1* | NM_057123 | 1.21 | 0.002 |
|  | caseinolytic peptidase X (E.coli) | *Clpx* | XM_217179 | 1.21 | 0.007 |
|  | proteasome (prosome, macropain) 26S subunit, non-ATPase, 8 | *Psmd8* | XM_214888 | 1.21 | 0.001 |
|  | proteasome (prosome, macropain) subunit, beta type 2 | *Psmb2* | NM_017284 | 1.20 | 0.001 |
|  | ubiquitin-conjugating enzyme E2M (UBC12 homolog, yeast) | *Ube2m* | XM_341790 | 1.20 | <0.001 |
|  | proteasome (prosome, macropain) subunit, alpha type 4 | *Psma4* | NM_017281 | 1.19 | 0.002 |
|  | Ubiquitin-conjugating enzyme E2 L3 (Ubiquitin-protein ligase L3) | *Ube2l3* | XM_226876 | 1.19 | 0.001 |
|  | protease, serine, 15 | *Prss15* | NM_133404 | 1.11 | 0.009 |
|  | ubiquitin specific protease 20 | *Usp20* | XM_231148 | -1.30 | 0.008 |
|  | ubiquitin-conjugating enzyme E2R 2 | *Ube2r2* | BF287028 | -1.40 | <0.001 |
| ***Translation*** | |  |  |  |  |
|  | polymerase (RNA) II (DNA directed) polypeptide I | *Polr2i* | XM_214895 | 1.46 | <0.001 |
|  | ribosomal protein L21 | *Rpl21* | NM_053330 | 1.38 | 0.001 |
|  | selective LIM binding factor, rat homolog | *Slb* | NM_053792 | 1.37 | <0.001 |
|  | ribosomal protein L26 | *Rpl26* | XM_225658 | 1.36 | <0.001 |
|  | ribosomal protein L33-like protein | *Rpl33* | AW144699 | 1.34 | 0.001 |
|  | DNA-directed RNA polymerases I, II, and III 7.0 kDa polypeptide | *Abc10a* | XM_219604 | 1.34 | 0.001 |
|  | ribosomal protein S26 | *Rps26* | XM_345933 | 1.34 | 0.001 |
|  | 60S ribosomal protein L32 | *Rpl32* | XM_345963 | 1.33 | 0.001 |
|  | ribosomal protein L22 | *Rpl22* | NM_031104 | 1.32 | 0.001 |
|  | large subunit ribosomal protein L36a | *Rpl36a* | XM_213224 | 1.32 | 0.001 |
|  | ribosomal protein L19 | *Rpl19* | XM_212869 | 1.32 | 0.001 |
|  | 40S ribosomal protein S27 | *Rps27* | XM_344908 | 1.31 | <0.001 |
|  | RNA polymerase II transcriptional coactivator | *Pol2* | XM_344312 | 1.31 | <0.001 |
|  | 40S ribosomal protein S7 | *Rps17* | XM_223834 | 1.31 | 0.001 |
|  | 40S ribosomal protein S25 | *Rps25* | XM_345662 | 1.31 | 0.002 |
|  | tRNA selenocysteine associated protein | *Secp43* | NM_023027 | 1.30 | <0.001 |
|  | ribosomal protein, large P2 | *Rplp2* | XM_344240 | 1.30 | 0.001 |
|  | ribosomal protein S24 | *Rps10* | XM_235376 | 1.30 | <0.001 |
|  | RPP20 protein | *Rpp20* | LOC288564 | 1.30 | <0.001 |
|  | ribosomal protein L27 | *Rpl27* | NM_022514 | 1.29 | <0.001 |
|  | Translationally controlled tumor protein | *Tctp* | XM_344072 | 1.29 | <0.001 |
|  | ribosomal protein S24 | *Rps24* | NM_031112 | 1.29 | 0.001 |
|  | ribosomal protein L7, cytosolic | *Rpl7* | LOC299934 | 1.26 | 0.001 |
|  | ribosomal protein S11 | *Rps11* | XM_344732 | 1.26 | 0.001 |
|  | 60S ribosomal protein L9 | *Rpl9* | XM_345559 | 1.26 | 0.001 |
|  | Dr1 associated protein 1 (negative cofactor 2 alpha) | *Drap1* | XM_215177 | 1.26 | <0.001 |
|  | polymerase (RNA) II (DNA directed) polypeptide F | *Polr2f* | NM_031335 | 1.25 | 0.003 |
|  | ribosomal protein L6 | *Rpl6* | XM_001062312 | 1.24 | 0.005 |
|  | polymerase (RNA) II (DNA directed) polypeptide C | *Polr2c* | XM_341643 | 1.24 | 0.001 |
|  | 60S ribosomal protein L23a | *Rpl23a* | XM_347346 | 1.24 | 0.007 |
|  | 60S acidic ribosomal protein P1 | *Rplp1* | XM_234147 | 1.23 | <0.001 |
|  | eukaryotic translation initiation factor 2, subunit 2 (beta) | *Eif2s2* | NM_199380 | 1.23 | 0.009 |
|  | 60S RIBOSOMAL PROTEIN L29 (P23) | *Rpl29* | XM_344416 | 1.23 | 0.001 |
|  | ribosomal protein S2 | *Rps2* | XM_343375 | 1.22 | 0.001 |
|  | mediator of RNA polymerase II transcription, subunit 31 homolog (yeast) | *Med31* | XM_213393 | 1.22 | 0.001 |
|  | 60S ribosomal protein L7a (Surfeit locus protein 3) (PLA-X polypeptide) | *Rpl7a* | XM_220311 | 1.22 | 0.010 |
|  | lysyl-tRNA synthetase | *Kars* | NM_001006967 | 1.20 | 0.001 |
|  | eukaryotic translation elongation factor 1 epsilon 1 | *Eif1e1* | XM_232665 | 1.20 | 0.019 |
|  | MON1 homolog b (yeast) | *Mon1* | TC472091 | 1.18 | <0.001 |
|  | threonyl-tRNA synthetase | *Tars* | NM_001006976 | 1.18 | 0.005 |
|  | eukaryotic translation initiation factor 4E member 2 | *Eif4el3* | XM_343616 | 1.17 | <0.001 |
|  | 40S ribosomal protein 20S protein | *Rps20* | XM_233420 | -1.25 | 0.002 |
|  | LIM homeobox protein 3 | *Lhx3* | AF370447 | -1.30 | 0.001 |
|  | 40S ribosomal protein S19 | *Rps19* | XM_223217 | -1.33 | 0.001 |
| ***Protein maturation*** | |  |  |  |  |
|  | kallikrein 8 (neuropsin/ovasin) | *Klk8* | XM_218648 | 1.77 | <0.001 |
|  | component of oligomeric golgi complex 1 | *Cog1* | XM_239373 | 1.69 | <0.001 |
|  | peptidyl arginine deiminase, type IV | *Padi4* | NM_017227 | 1.52 | <0.001 |
|  | hect (homologous to the E6-AP (UBE3A) carboxyl terminus) domain and RCC1 (CHC1)-like domain (RLD) 1 | *Herc1* | XM_236362 | 1.51 | <0.001 |
|  | DnaJ (Hsp40) homolog, subfamily B, member 3 | *Ugt1a6* | BF550847 | 1.51 | <0.001 |
|  | OMA1 homolog, zinc metallopeptidase (S. cerevisiae) | *Oma1* | XM_216446 | 1.44 | 0.001 |
|  | SEC61, gamma subunit | *Sec61g* | XM_346040 | 1.42 | <0.001 |
|  | suppressor of Ty 5 homolog (S. cerevisiae) | *Supt5h* | XM_341814 | 1.41 | <0.001 |
|  | 1-acylglycerol-3-phosphate O-acyltransferase 4 (lysophosphatidic acid acyltransferase, delta) | *Agpat4* | NM_133406 | 1.36 | <0.001 |
|  | peptidyl prolyl isomerase H | *Ppih* | XM_345576 | 1.36 | <0.001 |
|  | 1-acylglycerol-3-phosphate O-acyltransferase 3 | *Agpat3* | XM_215367 | 1.34 | <0.001 |
|  | phosphohistidine phosphatase 1 | *Phpt1* | XM_216013 | 1.32 | <0.001 |
|  | asparagine-linked glycosylation 5 homolog (yeast, dolichyl-phosphate beta-glucosyltransferase) | *Alg5* | AI012780 | 1.29 | <0.001 |
|  | signal recognition particle 19 | *Srp19* | XM_214599 | 1.29 | <0.001 |
|  | COP9 (constitutive photomorphogenic) homolog, subunit 6 (Arabidopsis thaliana) | *Cops6* | XM_222002 | 1.28 | <0.001 |
|  | DnaJ (Hsp40) homolog, subfamily C, member 8 | *Dnajc8* | XM_219742 | 1.27 | <0.001 |
|  | Sec61 beta subunit | *Sec61b* | XM_216400 | 1.26 | 0.001 |
|  | signal sequence receptor 4 | *Ssr4* | NM_017199 | 1.26 | <0.001 |
|  | DnaJ (Hsp40) homolog, subfamily C, member 15 | *Dnajd1* | XM_214238 | 1.26 | 0.001 |
|  | arsenic (+3 oxidation state) methyltransferase | *Cyt19* | NM_080890 | 1.25 | 0.001 |
|  | leucine aminopeptidase 3 | *Lap3* | CB547318 | 1.24 | 0.001 |
|  | XLas protein | *Xlas* | NM_021845 | 1.24 | <0.001 |
|  | peptidylprolyl isomerase B | *Ppib* | NM_022536 | 1.23 | <0.001 |
|  | DnaJ homolog subfamily B member 6 (Heat shock protein J2) | *Dnajb6* | XM_342607 | 1.19 | 0.004 |
|  | aminopeptidase puromycin sensitive | *Npepps* | XM_340889 | 1.17 | <0.001 |
|  | Glutamyl aminopeptidase (EAP) (Aminopeptidase A) | *Apa* | TC464630 | 1.14 | 0.007 |
|  | HemK methyltransferase family member 1 | *Hemk1* | XM_217259 | 1.14 | 0.016 |
|  | catechol-O-methyltransferase | *Comt* | AA858639 | -1.18 | 0.001 |
|  | proprotein convertase subtilisin/kexin type 1 inhibitor | *Pcsk1n* | NM_019279 | -1.19 | 0.001 |
|  | alanyl (membrane) aminopeptidase | *Anpep* | AF039891 | -1.23 | <0.001 |
|  | suppressor of Ty 3 homolog (S. cerevisiae) | *Supt3h* | BF524978 | -1.29 | 0.001 |
|  | arginine N-methyltransferase p82 isoform | *Prmt82* | BE111755 | -1.31 | <0.001 |
| ***Miscellaneous*** | |  |  |  |  |
|  | biogenesis of lysosome-related organelles complex-1, subunit 2 | *Bloc1s2* | XM_215245 | 1.29 | <0.001 |
|  | N-acetylgalactosamine kinase (GalNAc kinase) (Galactokinase 2) | *Galk2* | XM_215827 | 1.29 | <0.001 |
|  | adaptor protein complex AP-1, sigma 1 | *Ap1s1* | AW914195 | 1.26 | <0.001 |
|  | adaptor-related protein complex 2, sigma 1 subunit | *Ap2s1* | NM_022952 | 1.25 | 0.001 |
|  | peptide chain release factor 3 | *Gspt2* | XM_234139 | 1.18 | 0.001 |
|  | adaptor-related protein complex 2, beta 1 subunit | *Ap2b1* | NM_080583 | 1.17 | 0.001 |
|  |  |  |  |  |  |
| **Transcription** | |  |  |  |  |
| ***Chromatin*** | |  |  |  |  |
|  | Chromodomain-helicase-DNA-binding protein 1 | *Chd1* | XM_220804 | 1.58 | <0.001 |
|  | germinal histone H4 gene | *Hist1h4i* | AW144474 | 1.41 | 0.001 |
|  | H1 histone family, member 0 | *H1f0* | NM_012578 | 1.39 | <0.001 |
|  | histone 2, H2aa | *Hist1h2ae* | XM_345254 | 1.37 | <0.001 |
|  | histone 2a | *H2a* | XM_225393 | 1.36 | <0.001 |
|  | small nuclear ribonucleoprotein D3 | *Snrpd3* | AW142122 | 1.30 | <0.001 |
|  | small nuclear ribonucleoprotein polypeptide G | *Snrpg* | XM_232769 | 1.30 | 0.001 |
|  | H2B histone family, member T | *Hist1h2bj* | XM_225374 | 1.29 | 0.006 |
|  | sin3-associated polypeptide, 18kDa | *Sap18* | XM_220934 | 1.28 | <0.001 |
|  | Small nuclear ribonucleoprotein F | *Snrpf* | XM_345814 | 1.28 | 0.001 |
|  | histone 1 | *H2ao* | XM_344599 | 1.27 | <0.001 |
|  | H2A histone family, member V isoform 1 | *H2afv* | XM_225655 | 1.27 | 0.001 |
|  | Histone H2A.1 | *H2a1* | NM_021840 | 1.27 | <0.001 |
|  | SWI/SNF related, matrix associated, actin dependent regulator of chromatin, subfamily a, member 3 | *Smarca3* | TC477396 | 1.25 | <0.001 |
|  | small nuclear ribonucleoprotein D1 | *Snrpd1* | XM_346026 | 1.22 | <0.001 |
|  | histone deacetylase 3 | *Hdac3* | NM_053448 | 1.20 | <0.001 |
|  | chromatin modifying protein 6 |  | XM_221726 | 1.20 | 0.001 |
|  | chromodomain helicase DNA binding protein 3 | *Chd3* | XM_220602 | 1.18 | 0.001 |
|  | histone 1a | *H1a* | XM_225330 | 1.18 | 0.003 |
|  | heterogeneous nuclear ribonucleoprotein A2/B1 | *Hnrpa2b1* | XM_342684 | 1.17 | 0.001 |
|  | Methyl-CpG-binding domain protein 2 (Methyl-CpG-binding protein MBD2) | *Mbd2* | XM_214544 | 1.15 | 0.001 |
|  | chromodomain helicase DNA binding protein 5 | *Chd5* | XM_243049 | 1.14 | 0.007 |
| ***mRNA metabolism*** | |  |  |  |  |
|  | AU RNA binding protein/enoyl-coenzyme A hydratase | *Auh* | XM_341497 | 1.48 | <0.001 |
|  | Serpine1 mRNA binding protein 1 | *Rda288* | XM_342705 | 1.31 | <0.001 |
|  | RNA binding motif protein 25 | *Rbm25* | XM_345703 | 1.29 | <0.001 |
|  | upstream of NRAS | *Unr* | NM_054006 | 1.25 | <0.001 |
|  | guanine nucleotide binding protein-like 2 (nucleolar) | *Gnl2* | XM_342911 | 1.23 | <0.001 |
|  | RNA binding motif protein, X chromosome retrogene | *Rbmxrt* | XM_226369 | 1.21 | 0.001 |
|  | cleavage and polyadenylation specific factor 5 | *Cpsf5* | XM_214640 | 1.20 | 0.001 |
| ***Transcription*** | |  |  |  |  |
|  | transcription elongation factor B (SIII), polypeptide 2 | *Tceb2* | NM_031129 | 1.41 | <0.001 |
|  | POU domain, class 4, transcription factor 3 | *Pou4f3* | XM_344675 | 1.37 | <0.001 |
|  | transcription elongation factor A (SII) 1 | *Tcea1* | XM_223814 | 1.34 | <0.001 |
|  | calmodulin-binding transcription activator 1 | *Camta1* | XM_342982 | 1.31 | <0.001 |
|  | signal transducer and activator of transcription 3 | *Stat3* | NM_012747 | 1.31 | <0.001 |
|  | metallothionein-I gene transcription activator | *Mtf1* | AW144706 | 1.31 | 0.001 |
|  | transcription elongation factor B (SIII), polypeptide 1 | *Tceb1* | NM_022593 | 1.31 | <0.001 |
|  | step II splicing factor SLU7 | *Slu7* | XM_220315 | 1.30 | <0.001 |
|  | TATA box binding protein-like 1 | *Tbpl1* | XM_214744 | 1.29 | <0.001 |
|  | general transcription factor IIH, polypeptide 5 | *Gtf2h5* | BC058501 | 1.28 | <0.001 |
|  | single-stranded DNA binding protein 1 | *Ssbp1* | NM_183328 | 1.26 | <0.001 |
|  | PHD finger protein 10 | *Phr10* | XM_214780 | 1.25 | <0.001 |
|  | ring finger protein 141 | *Rnf141* | NM_001001800 | 1.24 | <0.001 |
|  | transcription factor EF1(A) | *M95791* | M95791 | 1.22 | <0.001 |
|  | Transcriptional regulator |  | TC482306 | 1.22 | 0.001 |
|  | ring finger protein 10 | *Rnf10* | XM_213797 | 1.21 | 0.026 |
|  | zinc finger and BTB domain containing 8 opposite strand | *Zbtb8* | XM_216347 | 1.20 | <0.001 |
|  | zinc finger, HIT domain containing 2 | *Znhit2* | XM_219526 | 1.15 | 0.001 |
|  | forkhead box K2 | *Foxk2* | XM_221212 | -1.44 | 0.001 |
|  | cofactor required for Sp1 transcriptional activation subunit 8 | *Crsp3* | CA509859 | -1.37 | <0.001 |
|  | zinc finger protein 238 | *Zfp238* | XM_345727 | -1.32 | 0.001 |
|  | zinc finger, CCHC domain containing 5 | *Zcchc5* | XM_228502 | -1.28 | 0.001 |
|  | zinc finger protein 294 | *Zfp294* | XM_213672 | -1.25 | 0.001 |
|  | zinc finger and BTB domain containing 5 | *Zbtb5* | BQ192627 | -1.23 | 0.005 |
|  | CXXC finger 1 (PHD domain) | *Cxxc1* | AW520826 | -1.21 | 0.002 |
|  | zinc finger protein, subfamily 1A, 5 | *Zfp1a5* | TC491880 | -1.18 | 0.008 |
| ***Miscellaneous*** | |  |  |  |  |
|  | Notch gene homolog 3 (Drosophila) | *Notch3* | NM_020087 | 1.59 | <0.001 |
|  | THAP domain containing 4 | *Thap4* | NM_001005564 | 1.49 | <0.001 |
|  | CCAAT/enhancer binding protein (C/EBP), gamma | *Cebpg* | XM_341842 | 1.30 | 0.003 |
|  | CCAAT/enhancer binding protein (C/EBP), delta | *Cebpd* | NM_013154 | 1.18 | 0.004 |
|  | Ppar binding protein, isoform 2 | *Pparbp* | TC470615 | 1.16 | <0.001 |
|  | period homolog 2 (Drosophila) | *Per2* | NM_031678 | -1.94 | <0.001 |
|  |  |  |  |  |  |
| **Cell turnover** | |  |  |  |  |
| ***Apoptosis*** | |  |  |  |  |
|  | deoxyribonuclease I | *Dnase1* | NM_013097 | 2.30 | <0.001 |
|  | PYD and CARD domain containing | *Pycard* | NM_172322 | 1.58 | <0.001 |
|  | BCL2-antagonist/killer 1 | *Bak1* | NM_053812 | 1.55 | <0.001 |
|  | STEAP family member 3 |  | NM_133314 | 1.41 | <0.001 |
|  | programmed cell death 8 | *Pdcd8* | NM_031356 | 1.33 | 0.001 |
|  | BAX protein, cytoplasmic isoform delta | *Bax* | AF235993 | 1.32 | <0.001 |
|  | cell division cycle 42 homolog (S. cerevisiae) | *Cdc42* | NM_171994 | 1.27 | 0.006 |
|  | apoptosis related protein APR-3; p18 protein | *Apr_3* | XM_216650 | 1.27 | 0.001 |
|  | TatD DNase domain containing 1 | *Tatdn1* | XM_228158 | 1.25 | <0.001 |
|  | programmed cell death 6 | *Pdcd6* | XM_217732 | 1.22 | <0.001 |
|  | caspase 7 | *Casp7* | NM_022260 | 1.21 | 0.001 |
|  | cell division cycle and apoptosis regulator 1 | *Ccar1* | XM_342143 | 1.18 | 0.001 |
|  | transmembrane BAX inhibitor motif containing 4 | *Tmbim4* | NM_199116 | 1.18 | 0.001 |
|  | CDC42 small effector 1 | *Cdc42se1* | AW920756 | 1.18 | 0.001 |
|  | CDC42 effector protein (Rho GTPase binding) 5 | *Cdc42ep5* | XM_341784 | 1.17 | 0.016 |
|  | Bcl2-associated athanogene 1 | *Bag1* | XM_216377 | 1.15 | 0.010 |
|  | BCL2/adenovirus E1B 19kDa-interacting protein 1 | *Bnip1* | NM_080897 | 1.15 | 0.001 |
|  | v-crk sarcoma virus CT10 oncogene homolog (avian) | *Crk* | BG671506 | 1.15 | 0.013 |
|  | apoptosis antagonizing transcription factor | *Aatf* | NM_053720 | 1.14 | 0.001 |
| ***Growth/ differentiation*** | |  |  |  |  |
|  | cyclin-dependent kinase inhibitor 3 | *Cdkn3* | XM_214152 | 1.48 | 0.007 |
|  | adipose differentiation-related protein | *ADRP* | AA874941 | 1.47 | 0.001 |
|  | bone morphogenetic protein 2 | *Bmp2* | BF559333 | 1.43 | <0.001 |
|  | c-myc binding protein | *Mycbp* | XM_216518 | 1.36 | <0.001 |
|  | growth factor, erv1 homolog (S. cerevisiae) | *Gfer* | NM_013222 | 1.30 | 0.001 |
|  | mago-nashi homolog, proliferation-associated (Drosophila) | *Magoh* | XM_216485 | 1.29 | <0.001 |
|  | death-associated kinase 2 | *Dapk2* | AW921062 | 1.27 | 0.001 |
|  | cyclin-dependent kinase-like 1 (CDC2-related kinase) | *Cdkl1* | XM_234266 | 1.26 | 0.011 |
|  | mm-Mago | *mago* | LOC298385 | 1.26 | <0.001 |
|  | HELICARD |  | CO396840 | 1.25 | 0.001 |
|  | DEAD (Asp-Glu-Ala-Asp) box polypeptide 10 | *Ddx10* | XM_236263 | 1.22 | 0.004 |
|  | endothelial differentiation, lysophosphatidic acid G-protein-coupled receptor, 2 | *Edg2* | NM_053936 | 1.21 | 0.005 |
|  | cyclin D3 | *Ccnd3* | NM_012766 | 1.20 | 0.002 |
|  | nerve growth factor receptor (TNFRSF16) associated protein 1 | *Ngfrap1* | NM_053401 | 1.19 | 0.001 |
|  | cyclin-dependent kinase 7 (homolog of Xenopus MO15 cdk-activating kinase) | *Cdk7* | XM_215467 | 1.19 | <0.001 |
|  | cyclin-dependent kinase 5 | *Cdk5* | NM_080885 | 1.18 | 0.021 |
|  |  |  |  |  |  |
| **Mucosal Barrier** | |  |  |  |  |
| ***Mucosal defense*** | |  |  |  |  |
|  | phospholipase A2, group IIA (platelets, synovial fluid) | *Pla2g2a* | NM_031598 | 3.73 | <0.001 |
|  | gastric mucin | *Muc* | XM_344685 | 1.70 | <0.001 |
|  | trefoil factor 3 | *Tff3* | NM_013042 | 1.70 | <0.001 |
|  | trefoil factor 1 | *Tff1* | NM_057129 | 1.64 | <0.001 |
|  | toll-like receptor 1 | *Tlr1* | XM_223421 | 1.49 | <0.001 |
|  | beta-2 microglobulin | *B2m* | NM_012512 | 1.40 | <0.001 |
|  | MHC class II region expressed gene KE2 | *Ke2* | NM_212506 | 1.36 | <0.001 |
|  | lectin, mannose-binding 2 | *Lman2* | XM_214428 | 1.25 | 0.001 |
| ***Immune response*** | |  |  |  |  |
|  | chemokine-like factor | *Cklf1* | NM_139111 | 1.33 | 0.001 |
|  | TRAF4 associated factor 1 | *Traf4af1* | NM_001004264 | 1.29 | 0.011 |
|  | protein-kinase, interferon-inducible double stranded RNA dependent inhibitor, repressor of (P58 repressor) | *Prkrir* | XM_218949 | 1.25 | 0.001 |
|  | interferon gamma inducible protein 30 | *Ifi30* | XM_214298 | 1.25 | <0.001 |
|  | host cell factor C1 regulator 1 (XPO1-dependent) | *Hpip* | AY245001 | 1.23 | 0.001 |
|  | CD320 antigen | *Cd320* | BF287508 | 1.22 | <0.001 |
|  | interferon induced transmembrane protein 2 (1-8D) | *Ifitm2* | NM_030833 | 1.22 | 0.001 |
|  | Tnf receptor-associated factor 2 | *Traf2* | XM_231032 | 1.20 | 0.001 |
|  | small inducible cytokine subfamily E, member 1 | *Scye1* | XM_342344 | 1.19 | <0.001 |
|  | T cell receptor | *Tcr* | L20997 | -1.36 | 0.001 |
| ***Miscellaneous*** | |  |  |  |  |
|  | basigin | *Bsg* | NM_012783 | 1.42 | <0.001 |
|  | high mobility group box 2 | *Hmgb2* | D84418 | 1.41 | 0.001 |
|  | metadherin | *Mtdh* | NM_133398 | 1.27 | <0.001 |
|  | High mobility group protein 1 | *Hmg1* | XM_344684 | 1.27 | <0.001 |
|  | claudin 23 | *Cldn23* | XM_224915 | 1.22 | 0.001 |
|  | Kruppel-like factor 4 (gut) | *Klf4* | NM_053713 | 1.21 | 0.001 |
|  | high mobility group nucleosomal binding domain 1 | *Hmgn1* | BI303604 | 1.11 | 0.156 |
|  | elastase 2, neutrophil | *Ela2* | CA507746 | -1.48 | 0.003 |
|  | myelin/oligodendrocyte glycoprotein alpha 4 isoform | *Moga4* | TC491669 | -1.27 | <0.001 |
|  | killer cell lectin-like receptor subfamily C, member 2 | *Klrc2* | NM_019261 | -1.24 | 0.001 |
|  | high mobility group protein 17 | *Hmgn2* | CA509211 | -1.16 | 0.002 |
|  | interferon stimulated exonuclease gene 20-like 1 | *Isg20l1* | XM_341874 | -1.16 | 0.024 |
|  |  |  |  |  |  |
| **Cytoskeleton** | |  |  |  |  |
|  | outer dense fiber of sperm tails 3-like 1 | *Odf3l1* | XM_236270 | 1.86 | <0.001 |
|  | myosin, light polypeptide 6, alkali, smooth muscle and non-muscle | *Myl6* | XM_343144 | 1.40 | <0.001 |
|  | tropomyosin 3, gamma | *Tpm3* | AW140804 | 1.38 | <0.001 |
|  | PDZ domain actin binding protein Shroom | *Shrm* | XM_344221 | 1.37 | <0.001 |
|  | tubulin cofactor a | *Tbca* | XM_345880 | 1.37 | <0.001 |
|  | myosin light chain 1 slow a | *Mlc1sa* | XM_222302 | 1.35 | 0.001 |
|  | Myosin light chain alkali, smooth-muscle isoform | *Mlc3sm* | XM_216335 | 1.35 | 0.001 |
|  | coactosin-like 1 (Dictyostelium) | *Cotl1* | XM_341700 | 1.35 | 0.001 |
|  | smooth muscle and non-muscle myosin alkali light chain isoform 2 | *Myl2* | XM_345356 | 1.30 | 0.001 |
|  | actin related protein 2/3 complex, subunit 5-like | *Arpc5* | XM_237111 | 1.29 | 0.001 |
|  | LIM and SH3 protein 1 | *Lasp1* | XM_346900 | 1.28 | 0.001 |
|  | fragile-X-related protein 1 isoform b | *Fxf1h9* | TC475930 | 1.28 | <0.001 |
|  | actin related protein 2/3 complex, subunit 3 | *Arpc3* | XM_213782 | 1.27 | 0.001 |
|  | outer dense fiber of sperm tails 2-like | *Odf2l* | XM_217691 | 1.27 | <0.001 |
|  | kinesin family member 5B | *Kif5b* | XM_341538 | 1.26 | <0.001 |
|  | kinesin family member 5B | *Kif5b* | XM_341538 | 1.24 | 0.001 |
|  | Ras-induced senescence 1 | *Ris1* | NM_057212 | 1.24 | 0.002 |
|  | Rho-associated coiled-coil forming kinase 1 | *Rock1* | NM_031098 | 1.23 | 0.008 |
|  | dynactin 3 | *Dctn3* | XM_218083 | 1.23 | 0.001 |
|  | Ran-interacting protein MOG1 | *Mog1* | XM_224238 | 1.23 | <0.001 |
|  | clathrin, light polypeptide (Lca) | *Clta* | NM_031974 | 1.23 | 0.001 |
|  | tropomodulin 3 | *Tmod3* | BF562779 | 1.21 | 0.001 |
|  | Keratin, type II cytoskeletal 8 (Cytokeratin 8) (Cytokeratin endo A) | *Ck8* | XM_213620 | 1.21 | 0.001 |
|  | RAS related protein 2a | *Rap2a* | NM_053741 | 1.20 | 0.001 |
|  | palladin |  | XM_214338 | 1.20 | 0.010 |
|  | myopalladin | *Mypn* | XM_228149 | -1.20 | 0.001 |
|  |  |  |  |  |  |
| **Transport** | |  |  |  |  |
|  | Retinoid binding protein 7 | *Rbp7* | XM_575960 | 3.97 | <0.001 |
|  | fatty acid binding protein 1, liver | *Fabp1* | NM_012556 | 2.90 | 0.001 |
|  | chloride channel calcium activated 6 | *Prp3* | NM_201419 | 2.47 | <0.001 |
|  | retinol binding protein 2, cellular | *Rbp2* | NM_012640 | 2.06 | <0.001 |
|  | aquaporin 3 | *Aqp3* | NM_031703 | 1.67 | <0.001 |
|  | aquaporin 7 | *Aqp7* | NM_019157 | 1.49 | <0.001 |
|  | apolipoprotein L2; apolipoprotein L-II | *Apol2* | XM_343282 | 1.46 | <0.001 |
|  | solute carrier family 27 (fatty acid transporter), member 4 | *Slc27a4* | TC483050 | 1.32 | <0.001 |
|  | potassium voltage gated channel, Shal-related family, member 2 | *Kcnd2* | NM_031730 | 1.32 | 0.001 |
|  | potassium intermediate/small conductance calcium-activated channel, subfamily N, member 4 | *Kcnn4* | NM_023021 | 1.26 | 0.001 |
|  | sodium channel associated protein 1 | *Sap1* | NM_153740 | 1.22 | 0.001 |
|  | ABC transporter ATP-binding protein |  | TC487705 | 1.22 | <0.001 |
|  | solute carrier family 22 (organic cation transporter), member 18 | *Slc22a18* | NM_001004260 | 1.21 | 0.001 |
|  | solute carrier family 22 (organic anion transporter), member 7 | *Slc22a7* | NM_053537 | 1.18 | 0.003 |
|  | solute carrier family 44, member 4 | *Ng22* | NM_212541 | 1.17 | 0.027 |
|  | arsA arsenite transporter, ATP-binding, homolog 1 (bacterial) | *Asna1* | XM_213848 | 1.13 | 0.063 |
|  | hyperpolarization-activated cyclic nucleotide-gated potassium channel 3 | *Hcn3* | NM_053685 | -1.15 | 0.002 |
|  | solute carrier family 6 (neurotransmitter transporter, noradrenalin), member 2 | *Slc6a2* | NM_031343 | -1.18 | <0.001 |
|  | solute carrier family 9 (sodium/hydrogen exchanger), member 5 | *Slc9a5* | NM_138858 | -1.22 | 0.001 |
|  | solute carrier family 4, member 1 | *Slc4a1* | BC085748 | -1.23 | 0.002 |
|  |  |  |  |  |  |
| **Oxidative stress** | |  |  |  |  |
|  | Metallothionein-2 | *Mt2* | BF556648 | 1.94 | <0.001 |
|  | metallothionein 1a | *Mt1a* | NM_138826 | 1.82 | <0.001 |
|  | microsomal glutathione S-transferase 3 | *Mgst3* | XM_213943 | 1.64 | <0.001 |
|  | glutathione S-transferase A3 | *Gsta5* | NM_031509 | 1.63 | 0.001 |
|  | Microsomal glutathione S-transferase 3 | *Gst3* | TC516234 | 1.62 | <0.001 |
|  | heme oxygenase (decycling) 1 | *Hmox1* | NM_012580 | 1.52 | 0.003 |
|  | epoxide hydrolase 2, cytoplasmic | Ephx2 | NM_022936 | 1.51 | 0.001 |
|  | glutathione-S-transferase, alpha type2 | *Gsta2* | NM_017013 | 1.38 | 0.002 |
|  | glutathione S-transferase omega 1 | *Gsto1* | NM_001007602 | 1.37 | <0.001 |
|  | superoxide dismutase 1 | *Sod1* | NM_017050 | 1.37 | 0.001 |
|  | glutathione S-transferase kappa 1 | *GST13-13* | NM_181371 | 1.36 | 0.001 |
|  | ATX1 (antioxidant protein 1) homolog 1 (yeast) | *Atox1* | NM_053359 | 1.31 | <0.001 |
|  | glutathione S-transferase, mu 5 | *Gstm5* | NM_172038 | 1.29 | 0.001 |
|  | glutathione peroxidase 1 | *Gpx1* | NM_030826 | 1.27 | 0.001 |
|  | paraoxonase 2 | *Pon2* | BC079462 | 1.14 | <0.001 |
|  | superoxide dismutase 3, extracellular | *Sod3* | NM_012880 | -1.41 | 0.001 |
|  |  |  |  |  |  |
| **Vescicle related** | |  |  |  |  |
|  | trafficking protein particle complex 1 | *Trappc1* | XM_213342 | 1.31 | <0.001 |
|  | Rab9 effector protein with kelch motifs | *Rabepk* | XM_216042 | 1.28 | <0.001 |
|  | vacuolar protein sorting 36 (yeast) | *Vps36* | XM_214382 | 1.23 | 0.001 |
|  | RAB27B, member RAS oncogene family | *Rab27b* | NM_053459 | 1.19 | 0.003 |
|  | Golgin 45 (Basic leucine zipper nuclear factor 1) | *Blzf1* | BC079114 | 1.19 | 0.004 |
|  | RAB8A, member RAS oncogene family | *Mel* | BC087584 | 1.18 | 0.002 |
|  | RAB5C, member RAS oncogene family | *Rab5c* | XM_213463 | 1.18 | 0.005 |
|  | vacuolar protein sorting 29 (S. pombe) | *Vps29* | XM_213780 | 1.16 | 0.002 |
|  | vesicle docking protein | *Vdp* | NM_019379 | 1.15 | <0.001 |
|  | trafficking protein, kinesin binding 2 | *Als2cr3* | AJ288898 | -1.26 | 0.002 |
|  |  |  |  |  |  |
| **Peptide hormones** | |  |  |  |  |
|  | Glucagon exon 6 | *Gcgex6* | K02813 | 2.56 | <0.001 |
|  | glucagon | *Gcg* | NM_012707 | 2.42 | <0.001 |
|  | pancreatic polypeptide | *Ppy* | NM_012626 | 1.71 | <0.001 |
|  | peptide YY | *Pyy* | XM_213468 | 1.64 | <0.001 |
|  | cholecystokinin | *Cck* | NM_012829 | 1.32 | 0.001 |
|  | insulin-like growth factor 1 | *Igf1* | AI169253 | -1.24 | 0.004 |
