# Supplementary material for: Impaired barrier function by dietary fructo-oligosaccharides (FOS) in rats is accompanied by increased colonic mitochondrial gene expression
Source: BMC Genomics. 2008 Mar 27;9:144. doi: 10.1186/1471-2164-9-144 (PMC2311291; doi:10.1186/1471-2164-9-144)
Supplement: Additional file 5 — FOS affected genes: Miscellaneous. Genes affected by FOS but not part of a significantly regulated biological process or genes with unknown function. [file 1471-2164-9-144-S5.doc]

**Additional file 5. Miscellaneous genes**

Genes affected by FOS but 1) not part of a significantly regulated biological process, or 2) genes with unknown function.

Genes included in this selection have a p-values<0.001 or are selected by RF.

|  | **Gene name** | **Gene symbol** | **Sequence ID** | **Fold change** | **P-value** |
| --- | --- | --- | --- | --- | --- |
| **Miscellaneous** | |  |  |  |  |
|  | Bq135360 |  | XM_214943 | 2.29 | 0.001 |
|  | Unknown |  | XM_213320 | 2.16 | <0.001 |
|  | all-trans-13,14-dihydroretinol saturase | *Rmt7* | NM_145084 | 2.10 | 0.008 |
|  | leucine rich repeat containing 48 | *Lrrc48* | XM_213320 | 2.08 | <0.001 |
|  | MEGF12 |  | BI289548 | 2.07 | 0.004 |
|  | Spetex-2C protein |  | XM_223916 | 1.95 | <0.001 |
|  | Yip1 domain family, member 3 |  | NM_001007801 | 1.94 | <0.001 |
|  | protein RAKd |  | XM_225541 | 1.92 | 0.002 |
|  | Unknown |  | XM_345942 | 1.88 | <0.001 |
|  | dipeptidase 1 (renal) | *Dpep1* | NM_053591 | 1.83 | 0.001 |
|  | zymogen granule protein 16 | *Zg16* | NM_134409 | 1.80 | <0.001 |
|  | Unknown |  | XM_344319 | 1.75 | <0.001 |
|  | Spetex-2F protein |  | XM_344320 | 1.73 | <0.001 |
|  | Unknown |  | ENSRNOT00000033720 | 1.71 | 0.001 |
|  | Spetex-2F protein |  | XM_229513 | 1.69 | <0.001 |
|  | Spetex-2C protein |  | XM_344470 | 1.65 | <0.001 |
|  | megakaryocyte stimulating factor |  | XM_217530 | 1.64 | <0.001 |
|  | neuregulin 2 | *Nrg2* | XM_214569 | 1.60 | <0.001 |
|  | phosphodiesterase 9A | *Pde9a* | NM_138543 | 1.59 | <0.001 |
|  | Spetex-2F protein |  | XM_223907 | 1.59 | <0.001 |
|  | Spetex-2F protein |  | XM_344291 | 1.57 | <0.001 |
|  | Spetex-2F protein |  | XM_224558 | 1.57 | <0.001 |
|  | Gm566 protein |  | XM_215660 | 1.57 | <0.001 |
|  | Spetex-2C protein |  | XM_344317 | 1.56 | <0.001 |
|  | Spetex-2C protein |  | XM_344320 | 1.56 | <0.001 |
|  | Unknown |  | XM_344320 | 1.56 | <0.001 |
|  | Ab2-225 |  | NM_001008770 | 1.55 | <0.001 |
|  | Unknown |  | AI029975 | 1.54 | 0.001 |
|  | proline-rich acidic protein 1 | *Prap1* | NM_031669 | 1.52 | <0.001 |
|  | Unknown |  | XM_345678 | 1.51 | <0.001 |
|  | von Willebrand factor | *Vwf* | XM_342759 | 1.50 | <0.001 |
|  | Unknown |  | XM_215236 | 1.50 | <0.001 |
|  | growth and transformation-dependent protein |  | XM_340993 | 1.49 | 0.001 |
|  | beta-microseminoprotein | *Msmb* | NM_019188 | 1.49 | 0.001 |
|  | Unknown |  | XM_213215 | 1.49 | 0.001 |
|  | Unknown |  | ENSRNOT00000024520 | 1.48 | <0.001 |
|  | Spetex-2F protein |  | XM_344467 | 1.48 | <0.001 |
|  | growth and transformation-dependent protein |  | XM_343890 | 1.47 | 0.001 |
|  | Unknown |  | NM_212516 | 1.46 | <0.001 |
|  | T-box 3 | *Tbx3* | NM_181638 | 1.46 | <0.001 |
|  | complement component 8, gamma polypeptide | *C8g* | XM_215990 | 1.46 | <0.001 |
|  | hexosaminidase B |  | XM_215455 | 1.45 | <0.001 |
|  | MARCKS-like 1 | *Mlp* | NM_030862 | 1.45 | <0.001 |
|  | Syntaxin 8 |  | TC505884 | 1.45 | <0.001 |
|  | tweety homolog 3 (Drosophila) | *Ttyh3* | AI548750 | 1.44 | <0.001 |
|  | Unknown |  | TC505453 | 1.44 | <0.001 |
|  | Unknown |  | BG665384 | 1.43 | <0.001 |
|  | Spetex-2F protein |  | XM_344295 | 1.43 | <0.001 |
|  | Unknown |  | XM_343501 | 1.43 | <0.001 |
|  | calmodulin 3 | *Calm3* | NM_012518 | 1.43 | <0.001 |
|  | Unknown |  | XM_341899 | 1.43 | <0.001 |
|  | Unknown |  | XM_213235 | 1.42 | <0.001 |
|  | Unknown |  | XM_223466 | 1.42 | <0.001 |
|  | Unknown |  | CA512222 | 1.42 | <0.001 |
|  | Unknown |  | XM_219527 | 1.42 | <0.001 |
|  | ceroid-lipofuscinosis, neuronal 6 | *Cln6* | XM_236325 | 1.42 | <0.001 |
|  | Unknown |  | AW918130 | 1.41 | <0.001 |
|  | Protein C11orf10 |  | XM_219574 | 1.41 | <0.001 |
|  | Unknown |  | XM_216787 | 1.41 | <0.001 |
|  | olfactory receptor 1305 | *Olr1305* | NM_001000959 | 1.41 | <0.001 |
|  | magnesium-dependent phosphatase-1 |  | XM_214200 | 1.41 | <0.001 |
|  | Unknown |  | XM_228601 | 1.40 | <0.001 |
|  | Unknown |  | XM_238484 | 1.40 | 0.001 |
|  | chromosome 6 open reading frame 188 |  | XM_215400 | 1.40 | 0.003 |
|  | Brain protein 44 |  | XM_213922 | 1.39 | <0.001 |
|  | Tceb2 protein |  | XM_224889 | 1.39 | <0.001 |
|  | Unknown |  | XM_216752 | 1.39 | 0.001 |
|  | Unknown |  | XM_216927 | 1.39 | 0.001 |
|  | Unknown |  | XM_235505 | 1.39 | <0.001 |
|  | cathepsin D | *Ctsd* | NM_134334 | 1.39 | 0.001 |
|  | Unknown |  | XM_217593 | 1.39 | <0.001 |
|  | Unknown |  | AY539890 | 1.39 | <0.001 |
|  | Unknown |  | XM_343571 | 1.39 | 0.005 |
|  | histidine triad nucleotide binding protein 1 | *Prkci* | NM_022192 | 1.39 | <0.001 |
|  | Unknown |  | XM_233756 | 1.38 | <0.001 |
|  | xanthine dehydrogenase | *Xdh* | NM_017154 | 1.38 | 0.005 |
|  | Unknown |  | AW140803 | 1.38 | <0.001 |
|  | Unknown |  | XM_218824 | 1.38 | <0.001 |
|  | Unknown |  | XM_345943 | 1.38 | <0.001 |
|  | Unknown |  | XM_221956 | 1.38 | 0.002 |
|  | protein kinase, cGMP-dependent, type 1 |  | XM_345021 | 1.38 | 0.001 |
|  | g20 protein |  | XM_236620 | 1.37 | <0.001 |
|  | Unknown |  | AY383676 | 1.37 | <0.001 |
|  | McKusick-Kaufman syndrome protein | *Mkks* | NM_001008353 | 1.37 | <0.001 |
|  | transaldolase 1 | *Taldo1* | NM_031811 | 1.37 | <0.001 |
|  | Unknown |  | XM_344860 | 1.37 | <0.001 |
|  | inhibitor of growth family, member 1 | *Ing1* | XM_225039 | 1.36 | <0.001 |
|  | SGT1, suppressor of G2 allele of SKP1 (S. cerevisiae) | *Sugt1* | XM_214242 | 1.36 | 0.001 |
|  | U2af1-rs2 |  | XM_217612 | 1.36 | 0.001 |
|  | hyaluronoglucosaminidase 2 | *Hyal2* | NM_172040 | 1.36 | 0.001 |
|  | BolA domain-containing protein like (11.4 kD) (1P25) |  | XM_216181 | 1.36 | <0.001 |
|  | RS21-C6 protein |  | XM_224631 | 1.36 | <0.001 |
|  | prenylated SNARE protein | *Ykt6* | NM_031692 | 1.36 | 0.002 |
|  | Unknown |  | AI070114 | 1.36 | 0.001 |
|  | Unknown |  | ENSRNOT00000038022 | 1.36 | <0.001 |
|  | Unknown |  | ENSRNOT00000037631 | 1.36 | 0.001 |
|  | Unknown |  | AW915978 | 1.36 | <0.001 |
|  | protein phosphatase 1, regulatory (inhibitor) subunit 14D | *Ppp1r14d* | NM_172011 | 1.36 | <0.001 |
|  | DUB-1 |  | XM_219062 | 1.36 | 0.002 |
|  | immature colon carcinoma transcript 1 | *Ict1* | XM_221110 | 1.36 | <0.001 |
|  | Unknown |  | AA924350 | 1.36 | <0.001 |
|  | Unknown |  | ENSRNOT00000036604 | 1.35 | <0.001 |
|  | histidine triad nucleotide binding protein 3 | *Hint4* | XM_341742 | 1.35 | <0.001 |
|  | Unknown |  | XM_238153 | 1.35 | 0.001 |
|  | surfeit 1 | *Surf1* | NM_172068 | 1.35 | 0.002 |
|  | Unknown |  | AW918967 | 1.34 | 0.001 |
|  | Unknown |  | ENSRNOT00000029812 | 1.34 | 0.001 |
|  | syntaxin 19 |  | ENSRNOT00000002658 | 1.34 | 0.003 |
|  | synaptotagmin-like 2 |  | XM_341881 | 1.34 | <0.001 |
|  | Unknown |  | XM_344912 | 1.34 | <0.001 |
|  | FK506 binding protein 3 | *Fkbp3* | XM_216717 | 1.34 | <0.001 |
|  | Unknown |  | NM_001008343 | 1.34 | <0.001 |
|  | Unknown |  | AY539887 | 1.34 | <0.001 |
|  | TGF beta-inducible nuclear protein 1; hairy cell leukemia protein 1 |  | XM_223612 | 1.33 | <0.001 |
|  | Unknown |  | XM_213467 | 1.33 | <0.001 |
|  | Unknown |  | XM_341604 | 1.33 | 0.001 |
|  | Unknown |  | AW917599 | 1.33 | <0.001 |
|  | Unknown |  | ENSRNOT00000037618 | 1.33 | <0.001 |
|  | coiled-coil domain containing 58 |  | XM_213612 | 1.33 | 0.001 |
|  | Unknown |  | XM_216368 | 1.33 | <0.001 |
|  | Unknown |  | AI236218 | 1.33 | 0.001 |
|  | Aig1 protein | *Aig1* | XM_214790 | 1.33 | <0.001 |
|  | Unknown | *Fam36a* | XM_213949 | 1.33 | <0.001 |
|  | calmodulin 3 | *Calm3* | NM_012518 | 1.33 | 0.001 |
|  | ferredoxin 1 | *Fdx1* | NM_017126 | 1.33 | <0.001 |
|  | Unknown |  | NM_001009275 | 1.33 | <0.001 |
|  | novel protein of unknown function (DUF423) family member |  | XM_213333 | 1.33 | <0.001 |
|  | olfactory receptor 1658 | *Olr1658* | NM_001000105 | 1.32 | <0.001 |
|  | chromosome 13 open reading frame 12 |  | XM_213700 | 1.32 | <0.001 |
|  | Unknown |  | XM_213347 | 1.32 | <0.001 |
|  | Unknown |  | BF565880 | 1.32 | <0.001 |
|  | Unknown |  | AY383680 | 1.32 | <0.001 |
|  | Unknown |  | XM_343793 | 1.32 | <0.001 |
|  | midnolin |  | TC480469 | 1.31 | 0.002 |
|  | malignant T cell amplified sequence 1 | *Mct1* | TC486673 | 1.31 | 0.002 |
|  | Unknown |  | XM_214998 | 1.31 | <0.001 |
|  | Unknown |  | BE113609 | 1.31 | 0.001 |
|  | Unknown |  | TC483660 | 1.31 | 0.001 |
|  | Unknown |  | XM_342957 | 1.31 | <0.001 |
|  | l-afadin |  | TC465067 | 1.31 | <0.001 |
|  | testis expressed gene 9 | *Tex9* | XM_217200 | 1.31 | 0.001 |
|  | putative phosphoinositide 5-phosphatase type II; C62 |  | BF522863 | 1.31 | <0.001 |
|  | upiquitin-like protein 8 |  | XM_345478 | 1.31 | <0.001 |
|  | membrane-spanning 4-domains, subfamily A, member 8B | *Ms4a8b* | XM_342026 | 1.31 | <0.001 |
|  | Unknown |  | AA946053 | 1.31 | <0.001 |
|  | Unknown |  | LOC308017 | 1.31 | <0.001 |
|  | Unknown |  | AW916609 | 1.31 | <0.001 |
|  | Unknown |  | XM_215378 | 1.31 | 0.001 |
|  | ubiquitously expressed transcript |  | NM_001006982 | 1.30 | <0.001 |
|  | nidogen-2 |  | XM_346140 | 1.30 | <0.001 |
|  | Unknown |  | XM_223828 | 1.30 | <0.001 |
|  | stromal membrane-associated protein 1 |  | AA956317 | 1.30 | 0.001 |
|  | Unknown |  | ENSRNOT00000033796 | 1.30 | <0.001 |
|  | ADP-ribosylation factor-like 6 | *Arl6* | XM_344009 | 1.30 | 0.002 |
|  | e(y)2 protein |  | XM_216913 | 1.30 | <0.001 |
|  | tumor necrosis factor receptor superfamily, member 12a | *Tnfrsf12a* | NM_181086 | 1.30 | <0.001 |
|  | Unknown |  | XM_342487 | 1.30 | 0.001 |
|  | histidine triad nucleotide binding protein 1 | *Hint1* | XM_231925 | 1.30 | <0.001 |
|  | Unknown |  | TC484487 | 1.30 | <0.001 |
|  | thioredoxin 1 | *Txn* | NM_053800 | 1.30 | 0.003 |
|  | brain abundant, membrane attached signal protein 1 | *Basp1* | NM_022300 | 1.30 | 0.001 |
|  | fucosidase, alpha-L- 1, tissue | *Fuca* | NM_012562 | 1.29 | 0.001 |
|  | mlrq-like protein |  | TC463415 | 1.29 | <0.001 |
|  | sorting nexing 24 | *Snx24* | NM_001008364 | 1.29 | 0.001 |
|  | headcase homolog (Drosophila) | *Heca* | AI137547 | 1.29 | <0.001 |
|  | HRAS like suppressor 3 | *Hrasls3* | NM_017060 | 1.29 | 0.001 |
|  | Unknown | *Rpe* | XM_237232 | 1.29 | <0.001 |
|  | Unknown |  | ENSRNOT00000030676 | 1.29 | 0.004 |
|  | Unknown |  | TC474661 | 1.29 | 0.001 |
|  | Unknown |  | XM_342099 | 1.29 | 0.001 |
|  | phytanoyl-CoA 2-hydroxylase 2 | *Hpcl2* | NM_053493 | 1.29 | <0.001 |
|  | Unknown |  | XM_222583 | 1.29 | 0.001 |
|  | Unknown |  | AW917232 | 1.29 | 0.005 |
|  | guanylate kinase 1 | *Guk1* | XM_220511 | 1.29 | <0.001 |
|  | P11 protein | *Cdtw1* | NM_134395 | 1.29 | 0.001 |
|  | DJ-1 protein |  | XM_344518 | 1.29 | <0.001 |
|  | Unknown |  | AY325138 | 1.29 | <0.001 |
|  | Unknown |  | XM_213403 | 1.28 | 0.005 |
|  | Unknown |  | XM_217080 | 1.28 | <0.001 |
|  | Unknown |  | TC485509 | 1.28 | <0.001 |
|  | stromal cell derived factor 4 | *Sdf4* | NM_130412 | 1.28 | 0.001 |
|  | Unknown |  | ENSRNOT00000033101 | 1.28 | 0.001 |
|  | Unknown |  | XM_217068 | 1.28 | 0.001 |
|  | S100 calcium-binding protein A14 (S114) | *S100a14* | TC476240 | 1.28 | 0.001 |
|  | carbonic anhydrase 1 | *Ca1* | XM_226922 | 1.28 | 0.007 |
|  | Ac1147 |  | XM_346230 | 1.28 | 0.001 |
|  | Unknown |  | ENSRNOT00000004085 | 1.28 | <0.001 |
|  | Usher syndrome 1C homolog (human) | *Ush1c* | NM_212521 | 1.28 | <0.001 |
|  | lysosomal-associated protein transmembrane 4B | *Laptm4b* | AA924846 | 1.28 | 0.006 |
|  | calcium/calmodulin-dependent protein kinase II, beta | *Camka2b* | TC476456 | 1.27 | <0.001 |
|  | Unknown |  | NM_001009635 | 1.27 | 0.001 |
|  | Unknown |  | XM_340892 | 1.27 | <0.001 |
|  | Unknown |  | BF565880 | 1.27 | <0.001 |
|  | Unknown |  | ENSRNOT00000030301 | 1.27 | <0.001 |
|  | Spetex-2C protein |  | XM_232690 | 1.27 | <0.001 |
|  | Unknown |  | ENSRNOT00000034494 | 1.27 | <0.001 |
|  | LSM5 homolog, U6 small nuclear RNA associated | *Lsm5* | XM_224630 | 1.27 | 0.001 |
|  | protein kinase inhibitor, gamma | *Pkig* | NM_153469 | 1.27 | <0.001 |
|  | transthyretin (4L369) |  | XM_215112 | 1.27 | 0.001 |
|  | adenine phosphoribosyl transferase | *Aprt* | XM_214704 | 1.27 | 0.001 |
|  | Unknown |  | XM_342482 | 1.27 | 0.001 |
|  | Unknown |  | TC494013 | 1.27 | 0.001 |
|  | Unknown | *Uqcrfs1* | XM_214457 | 1.27 | <0.001 |
|  | Unknown |  | TC481476 | 1.26 | <0.001 |
|  | tumor protein, translationally-controlled 1 |  | XM_341403 | 1.26 | <0.001 |
|  | enolase 1, alpha | *Eno1* | NM_012554 | 1.26 | 0.001 |
|  | XRCC1 DNA repair gene | *Xrcc1* | AW141286 | 1.26 | <0.001 |
|  | intersex-like (Drosophila) |  | XM_214868 | 1.26 | 0.007 |
|  | Unknown |  | XM_214121 | 1.26 | <0.001 |
|  | Unknown |  | XM_219819 | 1.26 | 0.025 |
|  | Unknown |  | ENSRNOT00000038574 | 1.26 | 0.001 |
|  | Unknown |  | TC498360 | 1.26 | 0.002 |
|  | prenylcysteine oxidase 1 | *Pcyox1* | NM_145085 | 1.26 | 0.001 |
|  | tumor protein, translationally-controlled 1 | *Tpt1* | NM_053867 | 1.26 | <0.001 |
|  | Unknown |  | ENSRNOT00000013882 | 1.26 | 0.014 |
|  | 3-5 exonuclease TREX1 |  | XM_345983 | 1.26 | 0.001 |
|  | olfactory receptor 1353 | *Olr1353* | NM_001000750 | 1.26 | 0.005 |
|  | Unknown |  | ENSRNOT00000030230 | 1.26 | 0.001 |
|  | fractured callus expressed transcript 1 | *Fxc1* | NM_053371 | 1.26 | 0.005 |
|  | Unknown |  | TC478611 | 1.26 | 0.001 |
|  | Nucleosome binding protein 1 (Nucleosome binding protein 45) (NBP-45) (GARP45 protein) |  | XM_346334 | 1.26 | 0.001 |
|  | Unknown |  | XM_215704 | 1.26 | <0.001 |
|  | Unknown |  | TC484496 | 1.26 | 0.001 |
|  | Unknown |  | XM_233725 | 1.26 | <0.001 |
|  | telomeric repeat binding factor 2 | *Terf2* | XM_341683 | 1.26 | 0.002 |
|  | TM2 domain containing 2 |  | XM_214374 | 1.26 | <0.001 |
|  | glyoxalase domain containing 1 |  | XM_233419 | 1.26 | 0.002 |
|  | guanine nucleotide binding protein, alpha q polypeptide | *Gnaq* | AW917479 | 1.25 | <0.001 |
|  | Unknown |  | XM_214293 | 1.25 | 0.001 |
|  | TGF beta-inducible nuclear protein 1; hairy cell leukemia protein 1 |  | XM_229208 | 1.25 | 0.002 |
|  | matrilin 2 | *Matn2* | BG664851 | 1.25 | 0.003 |
|  | phosphorylated adaptor for RNA export |  | NM_173133 | 1.25 | <0.001 |
|  | carbonic reductase 4 | *Cbr4* | NM_182672 | 1.25 | 0.004 |
|  | Unknown |  | ENSRNOT00000024085 | 1.25 | 0.001 |
|  | Parkinson disease (autosomal recessive, early onset) 7 | *Park7* | NM_057143 | 1.25 | <0.001 |
|  | FLI-LRR associated protein-1 |  | BC081883 | 1.25 | 0.001 |
|  | Unknown |  | ENSRNOT00000021215 | 1.25 | <0.001 |
|  | chromosome 14 open reading frame 138 |  | XM_343073 | 1.25 | <0.001 |
|  | beta-catenin-interacting protein ICAT | *Icat* | AA944531 | 1.25 | 0.001 |
|  | gem (nuclear organelle) associated protein 7 |  | AW919019 | 1.24 | 0.001 |
|  | Unknown |  | BF551664 | 1.24 | 0.001 |
|  | Ufm1-conjugating enzyme 1 | *Ufc1* | NM_001003709 | 1.24 | <0.001 |
|  | processing of precursor 7, ribonuclease P family, (S. cerevisiae) | *Pop7* | XM_213733 | 1.24 | 0.001 |
|  | Unknown |  | AW143153 | 1.24 | <0.001 |
|  | Unknown |  | XM_236996 | 1.24 | 0.001 |
|  | Unknown |  | LOC308017 | 1.24 | <0.001 |
|  | LSM3 homolog, U6 small nuclear RNA associated (S. cerevisiae) | *Lsm3* | XM_216220 | 1.24 | 0.001 |
|  | N-acetylneuraminic acid phosphatase | *Hdhd4* | XM_230779 | 1.24 | 0.001 |
|  | tumor protein, translationally-controlled 1 | *Tpt1* | NM_053867 | 1.24 | <0.001 |
|  | thioredoxin-like 2 | *Txnl2* | NM_032614 | 1.24 | <0.001 |
|  | EF hand domain family A1 | *Efha1* | BG665395 | 1.24 | <0.001 |
|  | Unknown |  | XM_341397 | 1.24 | 0.001 |
|  | Csr1 | *Csr1* | TC481205 | 1.23 | 0.001 |
|  | unknown |  | XM_225447 | 1.23 | 0.005 |
|  | RWD domain containing 4A |  | XM_214349 | 1.23 | 0.001 |
|  | Unknown |  | XM_215787 | 1.23 | <0.001 |
|  | GCD14/PCMT domain containing protein RGD1359191 |  | NM_001007706 | 1.23 | 0.015 |
|  | Unknown |  | NM_001004251 | 1.23 | <0.001 |
|  | Bat4 gene | *Bat4* | NR_002153 | 1.23 | 0.001 |
|  | protein phosphatase 1, regulatory (inhibitor) subunit 7 | *Ppp1r7* | NM_001009825 | 1.23 | 0.001 |
|  | Unknown |  | XM_228557 | 1.23 | 0.001 |
|  | t-complex testis expressed 1 | *Tctex1* | NM_031318 | 1.23 | 0.005 |
|  | Unknown |  | TC485289 | 1.22 | <0.001 |
|  | exosome component 3 | *Exosc3* | XM_233001 | 1.22 | 0.001 |
|  | nucleolar and coiled-body phosphoprotein 1 | *Nolc1* | NM_022869 | 1.22 | 0.001 |
|  | px19-like protein |  | NM_001009636 | 1.22 | 0.009 |
|  | Unknown |  | XM_214409 | 1.22 | <0.001 |
|  | deoxynucleotidyltransferase, terminal, interacting protein 1 | *Dnttip1* | NM_134400 | 1.22 | 0.007 |
|  | tetratricopeptide repeat domain 1 | *Ttc1* | NM_001005529 | 1.22 | <0.001 |
|  | Unknown |  | BF289611 | 1.22 | 0.001 |
|  | Unknown |  | XM_342549 | 1.22 | <0.001 |
|  | phosphatidylinositol glycan, class K |  | XM_215723 | 1.22 | 0.001 |
|  | maternal G10 transcript | *G10* | NM_053556 | 1.22 | <0.001 |
|  | Unknown |  | AW921285 | 1.22 | <0.001 |
|  | hydroxysteroid (17-beta) dehydrogenase 8 | *Hsd17b8* | NM_212529 | 1.22 | 0.001 |
|  | Unknown |  | NM_001005552 | 1.22 | 0.001 |
|  | bisphosphate 3'-nucleotidase 1 | *Bpnt1* | NM_171990 | 1.22 | <0.001 |
|  | islet cell autoantigen 1 | *Ica1* | NM_030844 | 1.22 | <0.001 |
|  | Unknown |  | AW915714 | 1.22 | <0.001 |
|  | heat shock 70kDa protein 14 | *Hspa14* | NM_001004257 | 1.22 | <0.001 |
|  | polyglutamine-containing protein |  | BF546374 | 1.22 | 0.001 |
|  | Unknown |  | XM_214983 | 1.22 | 0.001 |
|  | Unknown |  | TC462256 | 1.21 | 0.008 |
|  | ficolin A | *Fcna* | NM_031348 | 1.21 | <0.001 |
|  | dehydrogenase/reductase (SDR family) member 7B |  | NM_001008507 | 1.21 | <0.001 |
|  | transducer of ErbB-2.1 | *Tob1* | NM_133317 | 1.21 | 0.001 |
|  | Unknown |  | CB547362 | 1.21 | 0.001 |
|  | Unknown | *Olr1712-ps* | XM_227913 | 1.21 | 0.001 |
|  | Unknown |  | BF564723 | 1.21 | 0.001 |
|  | cGMP-dependent protein kinase 1, beta isozyme (CGK 1 beta) |  | XM_219807 | 1.21 | 0.010 |
|  | SUB1 homolog (S. cerevisiae) | *Rpo2tc1* | NM_001009618 | 1.21 | 0.001 |
|  | Unknown |  | TC511550 | 1.21 | 0.003 |
|  | transmembrane protein 41a | *Tm41a* | AW144067 | 1.21 | <0.001 |
|  | Unknown |  | XM_215797 | 1.21 | 0.003 |
|  | regulatory factor X-associated protein |  | CO806288 | 1.21 | 0.010 |
|  | Unknown |  | XM_234506 | 1.21 | 0.014 |
|  | discs, large homolog 3 (Drosophila) | *Dlgh3* | AW915015 | 1.21 | 0.001 |
|  | EF hand domain containing 2 | *Efha* | AI179527 | 1.21 | 0.002 |
|  | ASF1 anti-silencing function 1 homolog A (S. cerevisiae) | *Asf1a* | XM_215389 | 1.21 | <0.001 |
|  | Unknown |  | BQ199985 | 1.21 | 0.001 |
|  | keratinocyte associated protein 2 | *Krtcap2* | XM_215616 | 1.21 | <0.001 |
|  | phosphoglucomutase 1 | *Pgm1* | XM_214047 | 1.20 | <0.001 |
|  | Ngfi-A binding protein 2 | *Nab2* | XM_235224 | 1.20 | <0.001 |
|  | Unknown |  | BF558849 | 1.20 | 0.007 |
|  | ATG16 autophagy related 16-like 1 (S. cerevisiae) |  | XM_343618 | 1.20 | 0.001 |
|  | uridine monophosphate kinase | *Uck2* | TC480486 | 1.20 | 0.007 |
|  | Unknown |  | XM_342482 | 1.20 | 0.003 |
|  | presenilin enhancer 2 homolog (C. elegans) |  | XM_214897 | 1.20 | 0.001 |
|  | splA/ryanodine receptor domain and SOCS box containing 3 | *Tce1* | XM_220230 | 1.20 | 0.003 |
|  | TMEM9 domain family, member B |  | XM_215038 | 1.20 | 0.004 |
|  | NIMA (never in mitosis gene a)-related expressed kinase 1 | *Nek1* | XM_214340 | 1.20 | 0.001 |
|  | Unknown |  | ENSRNOT00000026141 | 1.20 | <0.001 |
|  | PDZ domain containing 11 |  | XM_217572 | 1.20 | 0.004 |
|  | chorionic somatomammotropin hormone 1-like 1 | *Csh1v* | NM_033233 | 1.20 | 0.001 |
|  | HS1 binding protein | *Hs1bp1* | NM_181627 | 1.20 | <0.001 |
|  | putative emu1 protein |  | XM_344282 | 1.20 | 0.003 |
|  | Y box protein 1 | *Nsep1* | NM_031563 | 1.19 | 0.004 |
|  | nucleotide binding protein 2 | *Nbp2* | XM_213239 | 1.19 | <0.001 |
|  | D-dopachrome tautomerase | *Ddt* | NM_024131 | 1.19 | 0.001 |
|  | nucleolar protein family A, member 3 |  | TC469721 | 1.19 | 0.001 |
|  | Basic FGF-repressed Zic-binding protein (mbFZb) | *Hiat1* | AW919172 | 1.19 | <0.001 |
|  | dehydrogenase/reductase (SDR family) member 4 | *Dhrs4* | NM_153315 | 1.19 | 0.002 |
|  | ADP-ribosylation factor 1 | *Arf1* | NM_022518 | 1.19 | 0.001 |
|  | Unknown |  | XM_342895 | 1.19 | 0.001 |
|  | phosphoglycerate mutase 1 | *Pgam1* | NM_053290 | 1.19 | 0.002 |
|  | necdin-like 2 | *Ndnl2* | XM_219708 | 1.19 | <0.001 |
|  | bradykinin receptor B1 | *Bdkrb1* | NM_030851 | 1.19 | 0.001 |
|  | progesterone receptor membrane component 1 | *Pgrmc1* | NM_021766 | 1.19 | 0.001 |
|  | cathepsin G | *Ctsg* | XM_214205 | 1.19 | 0.005 |
|  | malignant T cell amplified sequence 1 |  | XM_217587 | 1.19 | 0.001 |
|  | hydroxymethylbilane synthase | *Hmbs* | NM_013168 | 1.18 | 0.001 |
|  | bromodomain containing 3 | *Brd3* | XM_342396 | 1.18 | <0.001 |
|  | Unknown |  | CB570338 | 1.18 | <0.001 |
|  | phosphodiesterase isoform |  | AF053097 | 1.18 | 0.005 |
|  | transmembrane protein 15 | *Tmem15* | XM_231121 | 1.18 | <0.001 |
|  | sepiapterin reductase |  | XM_216190 | 1.18 | 0.002 |
|  | Unknown |  | BE118392 | 1.18 | 0.004 |
|  | HS1 binding protein | *Hs1bp1* | XM_001054758 | 1.18 | <0.001 |
|  | Unknown |  | XM_214819 | 1.18 | <0.001 |
|  | Unknown |  | XM_236221 | 1.17 | 0.002 |
|  | muted homolog (mouse) | *Muted* | XM_225255 | 1.17 | 0.006 |
|  | peroxisomal biogenesis factor 11A | *Pex11a* | NM_053487 | 1.17 | 0.007 |
|  | guanosine monophosphate reductase 2 | *Gmpr2* | XM_214203 | 1.17 | 0.003 |
|  | Unknown |  | TC470442 | 1.17 | 0.001 |
|  | Unknown |  | TC498834 | 1.17 | 0.022 |
|  | LAS1-like (S. cerevisiae) |  | XM_216095 | 1.17 | 0.004 |
|  | Unknown |  | AABR03055881 | 1.17 | 0.001 |
|  | protein phosphatase 1, regulatory (inhibitor) subunit 2 | *Ppp1r2* | NM_138823 | 1.17 | 0.036 |
|  | Down syndrome cell adhesion molecule | *Dscam* | NM_133587 | 1.17 | 0.007 |
|  | Unknown |  | XM_214202 | 1.17 | 0.002 |
|  | serine (or cysteine) peptidase inhibitor, clade B, member 5 | *Serpinb5* | NM_057108 | 1.17 | 0.015 |
|  | thioredoxin-like 1 | *Txnl1* | NM_080887 | 1.17 | 0.001 |
|  | arginine/serine-rich coiled-coil 1 |  | XM_347012 | 1.17 | 0.007 |
|  | heat shock 70kDa protein 9A | *Hspa9a* | XM_214583 | 1.17 | <0.001 |
|  | huntingtin interacting protein 2 | *Hip2* | XM_214043 | 1.17 | 0.001 |
|  | Unknown |  | TC478869 | 1.16 | 0.004 |
|  | myocardial ischemic preconditioning upregulated protein 2 |  | BF289772 | 1.16 | 0.005 |
|  | mitsugumin 29 | *Mg29* | XM_342316 | 1.16 | 0.012 |
|  | HIV-1 Rev binding protein 2 | *Hrb2* | XM_235128 | 1.16 | 0.007 |
|  | beta catenin-like 1 |  | XM_215906 | 1.16 | 0.010 |
|  | bone morphogenic protein receptor, type II (serine/threonine kinase) | *Bmpr2* | XM_217409 | 1.16 | <0.001 |
|  | chromosome 7 open reading frame 11 |  | AA850031 | 1.15 | 0.004 |
|  | olfactory receptor 380 | *Olr380* | NM_001001277 | 1.15 | 0.004 |
|  | Unknown |  | BF558804 | 1.15 | 0.005 |
|  | Unknown |  | AI228230 | 1.15 | 0.001 |
|  | tetraspanin 6 |  | BC086430 | 1.15 | 0.007 |
|  | Unknown |  | AW531732 | 1.15 | 0.041 |
|  | protein phosphatase 1, regulatory (inhibitor) 5 subunit 14B |  | XM_344123 | 1.15 | 0.101 |
|  | protein phosphatase 4, catalytic subunit | *Ppp4c* | XM_341929 | 1.14 | 0.007 |
|  | autocrine motility factor receptor | *Amfr* | AW917145 | 1.14 | 0.004 |
|  | constitutive photomorphogenic protein 1 |  | XM_341137 | 1.14 | 0.042 |
|  | heat shock protein 70kDa 12B | *Hspa12b* | XM_230610 | 1.13 | 0.001 |
|  | structure specific recognition protein 1 | *Ssrp1* | NM_031121 | 1.13 | 0.015 |
|  | uronyl-2-sulfotransferase | *Ust* | XM_341728 | 1.12 | 0.074 |
|  | Unknown |  | XM_215991 | 1.12 | 0.015 |
|  | protein phosphatase 1, catalytic subunit, gamma isoform | *Ppp1cc* | NM_022498 | 1.12 | 0.006 |
|  | Unknown |  | XM_214719 | 1.12 | 0.021 |
|  | polypyrimidine tract binding protein 1 | *Ptbp1* | BC061858 | 1.12 | 0.011 |
|  | Beta-sarcoglycan (Beta-SG) (43 kDa dystrophin-associated glycoprotein) (43DAG) | *Sgcb* | XM_223355 | 1.11 | 0.096 |
|  | paraspeckle protein 1 | *Pspc1* | XM_224234 | 1.11 | 0.026 |
|  | probable nocturnin protein |  | XM_344988 | 1.11 | 0.184 |
|  | WW domain binding protein 4 | *Wbp4* | XM_341360 | 1.11 | 0.003 |
|  | Unknown |  | TC503780 | -1.57 | 0.001 |
|  | vitamin A-deficient testicular protein 3-like |  | AF473843 | -1.51 | <0.001 |
|  | olfactory receptor 390 | *Olr390* | NM_001000558 | -1.48 | <0.001 |
|  | Unknown |  | TC511025 | -1.47 | <0.001 |
|  | Unknown |  | BF561025 | -1.47 | 0.002 |
|  | olfactory receptor 602 | *Olr602* | NM_001000333 | -1.44 | 0.002 |
|  | Unknown |  | BI283970 | -1.44 | 0.004 |
|  | beta-1,3-glucuronyltransferase 1 (glucuronosyltransferase P) | *B3gat1* | NM_054003 | -1.44 | <0.001 |
|  | Unknown |  | BG379104 | -1.43 | 0.002 |
|  | Unknown |  | TC502894 | -1.40 | <0.001 |
|  | coagulation factor II (thrombin) receptor-like 1 | *F2rl1* | NM_053897 | -1.40 | 0.001 |
|  | Discs large homolog 5 (Placenta and prostate DLG) (Discs large protein P-dlg) |  | XM_346168 | -1.40 | 0.007 |
|  | O-linked N-acetylglucosamine (GlcNAc) transferase (UDP-N-acetylglucosamine:polypeptide-N-acetylglucosaminyl transferase) | *Ogt* | NM_017107 | -1.39 | 0.016 |
|  | olfactory receptor 323 | *Olr323* | NM_001000245 | -1.38 | <0.001 |
|  | Fnbp1; rapostlin | *Fnbp1* | AA925321 | -1.36 | <0.001 |
|  | stomatin | *Stom* | AW914950 | -1.36 | <0.001 |
|  | Unknown |  | TC497359 | -1.36 | 0.001 |
|  | AXL receptor tyrosine kinase | *Axl* | BG665481 | -1.34 | <0.001 |
|  | ectonucleoside triphosphate diphosphohydrolase 4 | *Entpd4* | XM_341346 | -1.34 | 0.011 |
|  | Unknown |  | AI029007 | -1.33 | <0.001 |
|  | Unknown |  | XM_343707 | -1.32 | 0.003 |
|  | Unknown |  | XM_220245 | -1.32 | 0.005 |
|  | cytochrome P450, family 2, subfamily j, polypeptide 9 | *Cyp2j9* | NM_175766 | -1.32 | 0.005 |
|  | histidine decarboxylase | *Hdc* | NM_017016 | -1.32 | 0.019 |
|  | Unknown |  | AY383710 | -1.32 | <0.001 |
|  | Unknown |  | XM_233728 | -1.32 | <0.001 |
|  | Unknown |  | XM_347085 | -1.31 | <0.001 |
|  | Unknown |  | AY387075 | -1.31 | <0.001 |
|  | RW1 protein |  | XM_237056 | -1.31 | <0.001 |
|  | son of sevenless homolog 2 (Drosophila) | *Sos1* | XM_234263 | -1.31 | 0.001 |
|  | Unknown |  | AA957193 | -1.31 | <0.001 |
|  | sperm associated antigen 8 | *Spag8* | XM_342826 | -1.30 | <0.001 |
|  | Unknown |  | XM_346359 | -1.30 | 0.001 |
|  | Unknown |  | BE109513 | -1.29 | <0.001 |
|  | wee1 tyrosine kinase | *Wee1* | D31838 | -1.29 | 0.002 |
|  | Unknown |  | XM_229650 | -1.29 | 0.016 |
|  | unc-5 homolog A (C. elegans) | *Unc5a* | NM_022206 | -1.29 | 0.001 |
|  | transmembrane 4 superfamily member 1 | *Tm4sf1* | XM_215576 | -1.29 | 0.003 |
|  | Unknown |  | AA998203 | -1.28 | <0.001 |
|  | thyroid peroxidase | *Tpo* | NM_019353 | -1.28 | 0.001 |
|  | olfactory receptor 1229 | *Olr1229* | NM_001000444 | -1.28 | 0.001 |
|  | Unknown |  | AI236975 | -1.28 | 0.012 |
|  | calbindin 2 | *Calb2* | NM_053988 | -1.28 | 0.001 |
|  | Unknown |  | AI233877 | -1.28 | <0.001 |
|  | MEGF12 |  | XM_215648 | -1.27 | 0.001 |
|  | VPS10 domain receptor |  | XM_219993 | -1.27 | 0.001 |
|  | Unknown |  | TC471785 | -1.27 | 0.001 |
|  | Unknown |  | TC499194 | -1.27 | <0.001 |
|  | Down syndrome cell adhesion molecule-like 1 | *Dscaml1* | XM_236203 | -1.27 | <0.001 |
|  | Unknown |  | XM_218503 | -1.26 | 0.001 |
|  | Unknown |  | A_44_P859795 | -1.26 | 0.001 |
|  | Protein Njmu-R1 | *Njmu* | AW916017 | -1.25 | 0.005 |
|  | Unknown |  | BG377969 | -1.25 | 0.001 |
|  | Unknown |  | TC473445 | -1.25 | 0.004 |
|  | olfactory receptor 11 | *Olr11* | NM_001000114 | -1.25 | 0.001 |
|  | Unknown |  | CA505934 | -1.25 | <0.001 |
|  | Unknown |  | AA891475 | -1.25 | 0.004 |
|  | Lysozyme (1,4-beta-N-acetylmuramidase). | *Lyc* | TC497755 | -1.25 | <0.001 |
|  | Unknown |  | XM_220725 | -1.25 | 0.005 |
|  | Protein C6orf78 homolog |  | XM_241691 | -1.25 | <0.001 |
|  | chromosome 21 open reading frame 29 |  | XM_345089 | -1.25 | 0.003 |
|  | Unknown |  | AW143273 | -1.25 | 0.002 |
|  | Unknown |  | XM_345670 | -1.24 | 0.003 |
|  | Unknown |  | TC467485 | -1.24 | 0.001 |
|  | Unknown |  | TC492772 | -1.24 | 0.003 |
|  | nodal homolog (mouse) |  | XM_228285 | -1.24 | 0.001 |
|  | LanC (bacterial lantibiotic synthetase component C)-like 2 | *Lancl2* | XM_342699 | -1.24 | 0.004 |
|  | leucine rich repeat containing 28 | *Lrrc28* | AI014085 | -1.23 | 0.002 |
|  | Unknown |  | AI502658 | -1.23 | <0.001 |
|  | Unknown |  | XM_216071 | -1.23 | 0.003 |
|  | Unknown |  | XM_231141 | -1.23 | 0.002 |
|  | Unknown |  | A_44_P661776 | -1.23 | 0.004 |
|  | family with sequence similarity 38, member A | *Fam38a* | XM_341708 | -1.23 | 0.001 |
|  | Unknown |  | TC515656 | -1.23 | 0.006 |
|  | LInner nuclear membrane protein Man1 (LEM domain containing protein 3) | *Man1* | AW913991 | -1.23 | 0.002 |
|  | LDL receptor adaptor protein |  | XM_345591 | -1.22 | 0.001 |
|  | Unknown |  | TC477730 | -1.22 | <0.001 |
|  | protein kinase C and casein kinase substrate in neurons 3 | *Pacsin3* | BI276352 | -1.22 | 0.001 |
|  | dystrophia myotonica-protein kinase | *Dm15* | XM_218411 | -1.22 | <0.001 |
|  | Unknown |  | AI031045 | -1.22 | 0.001 |
|  | leukotriene B4 receptor | *Ltb4r* | NM_021656 | -1.22 | 0.004 |
|  | tripartite motif protein 27 | *Trim27* | BF417071 | -1.22 | 0.005 |
|  | Rsb-66 protein | *Rsb66* | NM_181694 | -1.22 | <0.001 |
|  | membrane glycoprotein |  | XM_341014 | -1.21 | 0.003 |
|  | Unknown |  | TC513827 | -1.21 | 0.001 |
|  | Unknown |  | ENSRNOT00000025841 | -1.21 | <0.001 |
|  | Unknown |  | AA819333 | -1.21 | 0.003 |
|  | phosphorylase kinase alpha 1 | *Phka1* | AA817929 | -1.21 | 0.036 |
|  | nuclear receptor subfamily 1, group D, member 2 | *Nr1d2* | NM_147210 | -1.20 | 0.053 |
|  | olfactory receptor 327 | *Olr327* | NM_001000249 | -1.20 | 0.001 |
|  | WW domain binding protein 11 |  | XM_224177 | -1.20 | 0.004 |
|  | Unknown |  | ENSRNOT00000004980 | -1.20 | 0.007 |
|  | Unknown |  | XM_230851 | -1.20 | 0.002 |
|  | Mpv17 protein | *Mpv17* | TC481399 | -1.19 | 0.002 |
|  | olfactory receptor 1750 | *Olr1750* | NM_212493 | -1.19 | <0.001 |
|  | synuclein, alpha interacting protein (synphilin) | *Sncaip* | XM_225768 | -1.19 | 0.057 |
|  | Unknown |  | AABR03025850 | -1.19 | 0.005 |
|  | nucleoredoxin | *Nxn* | CB546220 | -1.19 | 0.012 |
|  | Unknown |  | XM_213736 | -1.19 | 0.003 |
|  | triggering receptor expressed on myeloid cells-like 1 |  | BF523163 | -1.18 | <0.001 |
|  | olfactory receptor 1126 | *Olr1126* | NM_001000880 | -1.18 | 0.006 |
|  | Unknown |  | AW144240 | -1.18 | 0.001 |
|  | olfactory receptor 226 | *Olfr41* | NM_031710 | -1.18 | 0.001 |
|  | Unknown |  | TC483345 | -1.18 | 0.003 |
|  | Ten-m3 |  | XM_224839 | -1.18 | 0.001 |
|  | PR domain containing 4 | *Prdm4* | NM_133312 | -1.18 | 0.001 |
|  | MARVEL (membrane-associating) domain containing 2 | *Mrvldc2* | XM_345145 | -1.17 | 0.025 |
|  | Unknown |  | AABR03055518 | -1.17 | 0.001 |
|  | amphoretin-induced gene and ORF | *Amigo* | NM_206881 | -1.17 | 0.001 |
|  | SPARC-related modular calcium binding protein 1 |  | AA850706 | -1.17 | 0.004 |
|  | Unknown |  | TC499964 | -1.16 | 0.010 |
|  | Unknown |  | TC513186 | -1.16 | 0.055 |
|  | Arg/Abl-interacting protein ArgBP2 | *Argbp2* | AF396458 | -1.15 | 0.034 |
|  | spinster-like protein |  | XM_341927 | -1.15 | 0.023 |
|  | Unknown |  | AW921244 | -1.15 | 0.005 |
|  | F-box only protein 3 isoform 1 | *Fbxo3* | AW915329 | -1.15 | 0.001 |
|  | Unknown |  | XM_222452 | -1.14 | 0.003 |
|  | Unknown |  | AI059663 | -1.13 | 0.017 |
|  | testicular haploid expressed gene product isoform 2 |  | BC087065 | -1.11 | 0.075 |
|  | BM88 antigen |  | XM_341959 | -1.09 | 0.034 |
