# Supplementary material for: Impaired barrier function by dietary fructo-oligosaccharides (FOS) in rats is accompanied by increased colonic mitochondrial gene expression
Source: BMC Genomics. 2008 Mar 27;9:144. doi: 10.1186/1471-2164-9-144 (PMC2311291; doi:10.1186/1471-2164-9-144)
Supplement: Additional file 6 — Primer sequences. Sequences of the primers used for Q-PCR analysis. [file 1471-2164-9-144-S6.doc]

**Supplemental table 1** Primer sequences of the primers used for Q-PCR analysis

| **Gene symbol** | **Gene ID** | **Sense** | **Anti-sense** | **Product**  **length** |
| --- | --- | --- | --- | --- |
| *ActB* | NM_031144 | CTTTCTACAATGAGCTGCGTGTG | GTCAGGATCTTCATGAGGTAGTCTGTC | 315 |
| *Akr1b8* | NM_173136 | TTCACTATTACAGGACCCCAAGATTAAAG | CGCTCAACTGGAAGTCAAAGACC | 170 |
| *Atp5i* | XM_001075306 | TGACTTACTCAGAGCCTCGATTAGC | AGCCAACAACACCACGTTTGC | 243 |
| *Cox7b* | NM_182819 | AGAAGACACCTACTTTCCATGACAAATATG | TTTAATGAGTACATGATTCTTTGACTTGGC | 250 |
| *Gcg* | NM_012707 | AGGGACCTTTACCAGTGATGTGAG | TTCACCAGCCAAGCAATGAATTCC | 75 |
| *Me1* | M30596 | CTTTCAGGCGTGACTTACAGTGTAG | CCCAGATAACTACCCTGAGGAAACC | 76 |
| *Ndufb9* | XM_216929 | CGAGTGCTACAAGGTTCCAGAATG | TGCGGTGCCTGTCTCTATGTG | 273 |
| *Nup37* | XM_216872 | ATCACTCGGTCCAGTTATCCTCAAG | AGCTTATGGTCTCCTCCAACTGC | 248 |
| *Pla2g2a* | NM_031598 | TTCTGACCTACAAGTTCTCCTACCG | TCAGCAACTGGGCGTCTTCC | 191 |
| *Plekha6* | XM_341118 | TCAATAAGGAGCTATCCACTCCAGAC | GCACAGGAGATTTCAATCCGCTTC | 250 |
| *Psma31* | BN000326 | AAGCTGCAAAGACAGAAATAGAAAAGC | TTGTCGTCATCTGATTCATCTTCTTCC | 250 |
| *Rbp7* | XM_575960 | CTGGGAGAACGACAAACTCACTTG | CCAATATAAGGCTCTTTATCAACCCAAAAC | 250 |
| *Mrps16* | XM_001064095 | CACTACCTAACAGTCATGGAGAAAAGC | CACTGAAGTCAGCTTGCTTCTGTC | 250 |
| *Sdhb* | XM_216558 | AGGCTTATCGCTGGATGATCGAC | GAAGGGACTCACGCCAGAGC | 250 |
| *Tff3* | NM_013042 | CACATCAGAGCAGTGTAACAACCG | GCTGACTGTAAGGTCTTTATTCTTCTGG | 250 |
| *Timm8b* | NM_022541 | TGGTGAAGCGGACGAAGCG | ATGGCAAGAGTAGTGTCAATGAATCG | 198 |
| *Uqcrb* | XM_001074024 | TGAGAGATGATACAATACCTGAAACTGAAG | TCTCCTTTCTTTCCCAAATAACCTCTTTC | 216 |
